# Wave Turbulence and Energy Cascade in the Hippocampus

**DOI:** 10.3389/fnsys.2018.00062

**Published:** 2019-01-04

**Authors:** Alex Sheremet, Yu Qin, Jack P. Kennedy, Yuchen Zhou, Andrew P. Maurer

**Affiliations:** ^1^Engineering School of Sustainable Infrastructure & Environment (ESSIE), University of Florida, Gainesville, FL, United States; ^2^Department of Neuroscience, McKnight Brain Institute, College of Medicine, University of Florida, Gainesville, FL, United States; ^3^Department of Biomedical Engineering, University of Florida, Gainesville, FL, United States

**Keywords:** hippocampus, mesoscopic collective action, theta-gamma coupling, kinetic equation, turbulence, spectral evolution, bispectrum analysis, self-organized criticality (SOC)

## Abstract

Mesoscale cortical activity can be defined as the organization of activity of large neuron populations into collective action, forming time-dependent patterns such as traveling waves. Although collective action may play an important role in the cross-scale integration of brain activity and in the emergence of cognitive behavior, a comprehensive formulation of the laws governing its dynamics is still lacking. Because collective action processes are macroscopic with respect to neuronal activity, these processes cannot be described directly with methods and models developed for the microscale (individual neurons).To identify the characteristic features of mesoscopic dynamics, and to lay the foundations for a theoretical description of mesoscopic activity in the hippocampus, we conduct a comprehensive examination of observational data of hippocampal local field potential (LFP) recordings. We use the strong correlation between rat running-speed and the LFP power to parameterize the energy input into the hippocampus, and show that both the power and non-linearity of collective action (e.g., theta and gamma rhythms) increase with increased speed. Our results show that collective-action dynamics are stochastic (the precise state of a single neuron is irrelevant), weakly non-linear, and weakly dissipative. These are the principles of the theory of weak turbulence. Therefore, we propose weak turbulence a theoretical framework for the description of mesoscopic activity in the hippocampus. The weak turbulence framework provides a complete description of the cross-scale energy exchange (the energy cascade). It uncovers the mechanism governing major features of LFP spectra and bispectra, such as the physical meaning of the exponent α of power-law LFP spectra (e.g., *f*^−α^, where *f* is the frequency), the strengthening of theta-gamma coupling with energy input into the hippocampus, as well as specific phase lags associated with their interaction. Remarkably, the weak turbulence framework is consistent with the theory of self organized criticality, which provides a simple explanation for the existence of the power-law background spectrum. Together with self-organized criticality, weak turbulence could provide a unifying approach to modeling the dynamics of mesoscopic activity.

## 1. Introduction

Hebb's (1958) hypothesis that no psychological function can be attributed uniquely to any segment of cortex has the profound implication that cognition emerges from the coordination of activity across all spatial and temporal scales of the brain (Lashley, 1958; Allen and Collins, 2013), and that understanding the brain begins with studying the nature and role of different scales of brain activity.

Temporal scales, defined based on the frequency structure of extracellular recordings (local-field potential, LFP), are typically more accessible to observations. Their relation to spatial scales is not exactly known, but they are assumed to be in a monotonic relation to spatial scales (lower frequencies correspond to larger populations; Buzsáki and Draguhn, 2004). The Fourier spectra of hippocampal LFP recordings cover a frequency range approximately from 0.05 to 500 Hz, with spectra generally decaying as a power law. Two scales ranges are readily identified. The high end of the spectrum, say, *f* > 200 Hz^1^ represents the microscale, mainly occupied by action potential activity of single neurons, fast synaptic time constants, ion channel opening and closing, and heat dissipation (energy sinks)^2^. These processes are generated by microscopic neural units such as individual neurons, pairs of excitatory/inhibitory neurons, or small neuronal sequences (e.g., Lorente de No, 1938). The low end of the frequency spectrum (say, *f* < 60 Hz) is assumed to represent macroscopic processes encompassing several segments of the brain. For example, the theta rhythm is observed across hippocampus, entorhinal cortex, hypothalamus, prefrontal cortex, and others; e.g., Vertes and Kocsis, 1997; Buzsaki, 2002; Siapas et al., 2005). It is associated with active exploration and REM sleep, and is assumed to provide the temporal structure for the organization of local networks (Green and Arduini, 1954; Green and Machne, 1955; Vanderwolf, 1969; Lisman and Idiart, 1995; Buzsaki, 2002).

In addition to these two scales, cortical activity exhibits an intermediate (meso-) scale (e.g., Freeman, 2000a; Lubenov and Siapas, 2009; Patel et al., 2012; Muller et al., 2018; Zhang et al., 2018), which spans the frequency band 60 < *f* < 200 Hz, a range of oscillations collectively referred to as the gamma rhythm (Buzsáki et al., 1983; Bragin et al., 1995; Belluscio et al., 2012; Lasztóczi and Klausberger, 2014; Schomburg et al., 2014). Mesoscopic processes have been observed within brain regions associated with higher cognition, such as the neocortex and the hippocampus, that exhibit anisotropic and homogeneous mesoscopic structure^3^. The monotonic relation between temporal and spatial scales places mesoscopic spatial scales in the order of a few millimeters in rats, suggesting that they represent *collective neuronal activity* (i.e., synchronized neuronal firing; Buzsáki and Draguhn, 2004)^4^. Rhythms in the 8–200 Hz range have the spatial structure of propagating perturbations, i.e., waves (Petsche and Stumpf, 1960; Lubenov and Siapas, 2009; Patel et al., 2012, 2013; Muller et al., 2018).

Collective action responds to task behavior and intensity of behavior activity. During spatial exploration, theta and gamma increase their power and develop measurable phase coupling in response to intensity of activity (Whishaw and Vanderwolf, 1973; Morris and Hagan, 1983; Bragin et al., 1995; Chorbak and Buzsaki, 1998; Chen et al., 2011; Ahmed and Mehta, 2012; Kemere et al., 2013; Sheremet et al., 2016; Zheng et al., 2016). This suggests a relation to cognition that is particularly intriguing, because isotropic and homogeneous mesoscopic neuronal structures are the type of environment that would favor collective action over that of microscopic elements. While mesoscopic collective activity could be explained away as a marginally-significant synchronization effect of strongly non-linear microscopic units (Buzsaki, 2006), its strong relation to isotropic and homogeneous networks and its connection to behavior suggest that it might be in fact *the function* of this type of network. Freeman and Vitiello (2010) hypothesize, citing Lashley (1942), that mesoscale processes reflect the essential cognition step of abstraction and generalization of a particular stimulus to a category of equivalent inputs, “because they require the *formation of non-local, very large-scale statistical ensembles* (our emphasis)”^5^.

*We therefore conjecture that mesoscopic action plays a crucial role in the integration of brain activity across scales, and that its stochastic character is essential for the fulfillment of this role*. This conjecture changes significantly the interpretation of LFP recordings and possibly other observations of of hippocampal activity. For example, rather than attributing special significance to the activity of microscopic elements (like musical scores played by separate instruments), LFP recordings should be interpreted as local observations of a stochastic process representing collective action by mesoscale ensembles involving a large number of microscopic units, in which the precise state of a single unit is irrelevant.

Despite a few brilliant insights into collective action dynamics (e.g., Wilson and Cowan, 1973; Wright and Liley, 1995; Freeman, 2000a, 2006, 2007; Freeman and Vitiello, 2010; Cowan et al., 2016), a consistent theoretical approach to mesoscopic dynamics is missing. It is important to note that, because of the scale separation, mesoscopic and microscopic dynamics are different, therefore the wealth of knowledge accumulated about microscopic physics cannot be directly extended to mesoscopic processes. The notion that theory is scale-dependent is common in physics: macroscopic systems are characterized by state variables and laws that are typically inaccessible directly to microscale theories. An example is Boltzmann's celebrated *H*-theorem, which introduces the entropy (*H*) as a new state variable, and elucidates the process through which time-reversible microscopic dynamics begets the macroscopic irreversibility of evolution toward equilibrium (Gibbs, 1902; Khinchin, 1949; Toda et al., 1983; Pathria and Beale, 2011)^6^.

Here, we propose the theory of weak turbulence as a framework for the description of mesoscopic collective action and its energy balance in the hippocampus. The paper is organized as follows:

*(i)* In section 2, we discuss briefly data collection and analysis procedures and provide some guidance on reading the bispectral maps used to estimate non-linear coupling between Fourier components of LFP recordings.

*(ii)* In section 3.1, we examine stochastic features LFP recordings, using second and third order correlators (spectra and bispectra). The goal of this section is to highlight important characteristics of collective action and their dependency on energy input into the hippocampus. This perspective is important because it captures the transformation (ultimately, the time evolution) of the LFP statistical measures, thus providing a dynamical view of mesoscopic activity. In particular, the evolution of energy distribution over spatial or temporal scales is relevant for describing the energy balance of collective action under non-linear interactions.

*(iii)* Section 3.5 introduces and discusses the theory of weak turbulence as a framework for mesoscopic collective action. Originally formulated for hydrodynamics, the theory of turbulence has expanded in scope through the work of Richardson, Kolmogorov, and Zakharov Richardson (1922), Zakharov et al. (1992), and Kolmogorov (1941) to become the theoretical foundation of physical disciplines ranging from plasma physics, to non-linear optics, Bose-Einstein condensation, water waves, coagulation-fragmentation processes, and many others (Zakharov et al., 1992; L'vov, 1998; Nazarenko, 2011). At the center of the turbulence theory is the study of internal energy processes in non-linear, multi-scale systems with a large number of components. The non-linear character of the system allows for scales to interact, creating the conditions for cross-scale flows of energy and other conserved quantities. Non-linearity implies interaction connects evolution across scales, allowing for a cross-scale flux of energy called the *turbulent cascade* (Richardson, 1922; Kolmogorov, 1941). The turbulent cascade is the fundamental property defining turbulence.

*(iv)* In section demonstrate the capabilities of the theory by using the three-wave equations (a simplified, universal non-linear interaction modeling framework) a to re-evaluate the significance of linear and non-linear structure of hippocampal LFP recordings.

*(v)* The significance of the turbulence theoretical framework for understanding collective action, and its implications for cognition, are discussed in section 4.

Further details of the weak turbulence formalism are given in the Supplementary Material.

## 2. Materials and Methods

### 2.1. Subjects and Behavioral Training

Rat r539♂-maurer used in this study belongs to a cohort of 4–9 months old Fisher344-Brown Norway Rats (Taconic; see e.g, Zhou et al. 2018 for additional information about the cohort). The methods detailing the collection for these rats have been described in detail elsewhere (Zhou et al., 2018). Briefly, following acclimation to the University of Florida colony, animals were trained to traverse a circular track for food reward (45 mg, unflavored dustless precision pellets; BioServ, New Jersey; Product #F0021). During this time, their body weight was slowly reduced to 85% to their ad libitum baseline. Rats were surgically implanted with a custom single shank silicon probe from NeuroNexus (Ann Arbor, MI), designed such that thirty-two recording sites spanned across multiple hippocampal lamina.For probe preparation instructions and surgical methods, see (Vandecasteele et al., 2012; Zhou et al., 2018).

All behavioral procedures were performed in accordance with the National Institutes of Health guidelines for rodents and with protocols approved by the University of Florida Institutional Animal Care and Use Committee.

### 2.2. Neurophysiology

Following recovery from surgery, the rat was retrained to run unidirectionally on a circle track (outer diameter: 115 cm, inner diameter: 88 cm), receiving food reward at a single location. Following a few days of circle track running, the rats were trained to run on a digital-8 maze (121 × 101 cm, L × W). During these sessions, the local-field potential was record on a Tucker-Davis Neurophysiology System (Alachua, FL) at ~ 24 kHz (PZ2 and RZ2, Tucker-Davis Technologies). The animals position was recorded at 30 frames/s with a spatial resolution <0.5 cm/pixel.

### 2.3. Spectral Analysis

The spectral analysis of the LFP in the current study was based on standard techniques used for stationary signals Priestley, 1981; Papoulis and Pillai, 2002. Descriptions of the stochastic estimators, meaning, normalization procedures, as well as how to interpret the bispectral maps, are given in Hasselmann et al. (1963), Rosenblatt and Van Ness (1965), Swami et al. (2001), Elgar (1987), Elgar and Guza (1985), and Sheremet et al. (2016), and many others. Here, we remind the reader the main definitions and terminology.

Assume the LFP recordings *g*(*t*) and *h*(*t*) are realizations of zero-mean stochastic processes, stationary in the relevant statistics, with Fourier transforms *G*(*f*_*n*_) and *H*(*f*_*n*_), *n* = 1, ⋯ , *N*. The second and third order spectral statistics are estimated using cross-spectrum and bispectrum, defined as

(1) $$S_{n}^{gh}=S^{gh}\left(f_{n}\right)=\langle G_{n}H_{n}^{*}\rangle ,$$

(2) $$B_{mn}=B\left(f_{m},f_{n}\right)=\langle G_{m}G_{n}G_{m+n}^{*}\rangle .$$

where the angular brackets denote the ensemble average, the asterisk denotes complex conjugation. We will generally omit the superscript when a single time series is involved. The diagonal $S_{n}^{g,g}$ of the cross-spectrum matrix are power spectra. The coherence and phase lag of time series *g* and *h* are the normalized modulus and phase of the cross-spectrum,

(3) $$C_{n}^{gh}=\frac{S^{gh}\left(f_{n}\right)}{\sqrt{S_{n}^{gg}S_{n}^{hh}}},\text{and }\Theta_{n}^{gh}=\arg S^{gh}\left(f_{n}\right)$$

The cross-spectrum matrix provides information about the degree of correlation and phase lags for between different time series; spectra describe the frequency distribution of the variance of processes *g* and *h*, i.e., a complete characterization of the average linear structure of the Fourier representation.

The bispectrum provides information about the phase correlations between different frequency components of the same time series (e.g., Sheremet et al., 2016; Kovach et al., 2018). The bispectrum is statistically zero if the Fourier coefficients are mutually independent, i.e., for a linear system, and will exhibit peaks at triads (*f*_*n*_, *f*_*m*_, *f*_*n* + *m*_) that are phase correlated. The real and imaginary part of the bispectrum are related to the skewness S (e.g., positive skewness corresponds to sharp barrow peaks and flat troughs) and asymmetry A (e.g., positive asymmetry corresponds to the front of the wave being steeper than the back, similar to a saw-tooth wave) of the time series *g* through

(4) $$\sigma^{-3}\sum_{m,n}B_{mn}=S+iA,$$

where σ is its standard deviation (Haubrich and MacKenzie, 1965; Masuda and Kuo, 1981). The bicoherence *b*_*mn*_ and biphase Φ_*mn*_ are defined in way similar to the coherence and phase lag as the normalized modulus and the argument of the bispectrum, that is

(5) $$\begin{array}{ll} b_{mn} & =\frac{B_{mn}}{\sqrt{S_{m}S_{n}S_{m+n}}},\text{ and }\Phi_{mn}=\arg B_{mn}. \end{array}$$

**Figure 1:**
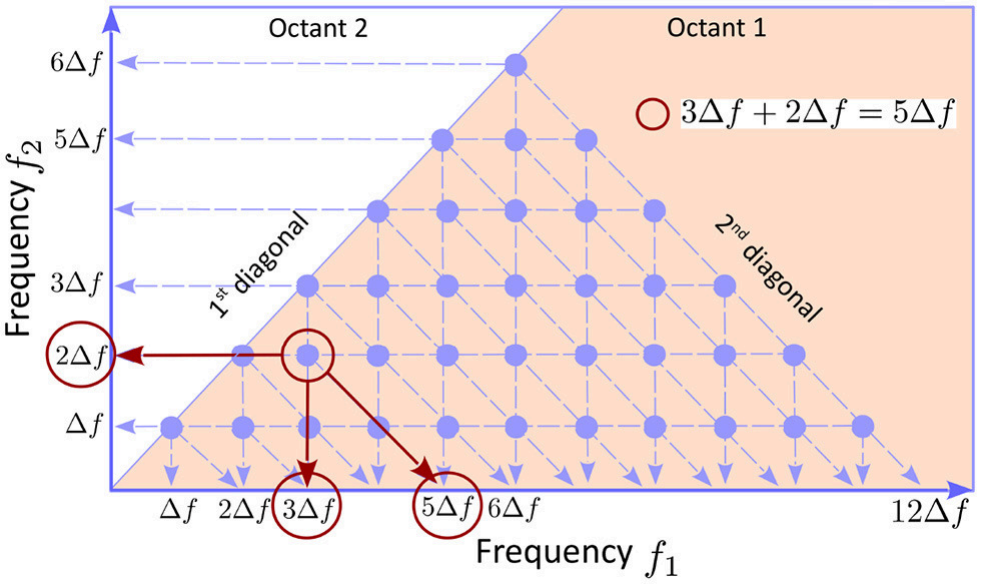
The underlying geometry of a bispectrum plot. The symmetries of the bispectrum (see text) imply that the smallest area in the plans (*f*_*n*_, *f*_*m*_) containing non-redundant information is the first octant, the solid angle between the the *f*_1_ axis and first diagonal. Because the bispectrum is computed using discretized frequencies, it is defined only at points (*f*_*m*_, *f*_*n*_) with *n, m* = 1, ⋯ , *N* (blue dots; *N* = 12 here). Because *f*_*m*_ + *f*_*n*_ ≤ *f*_*N*_, the triads of the type $G_{n}G_{m}G_{m+n}^{*}$ can only be constructed for *m* + *n* ≤ *N*, hence the triangular shape of the bispectral domain. If the bicoherence exhibits a peak (e.g., the red circle) the triad of modes that are phase coupled can be identified as the coordinates of the peak, together with the intersection between the horizontal axis and a parallel to the second diagonal passing through the peak (red arrows).

The bispectrum definition 2 has the following symmetries: 1) *B*_*mn*_ = *B*_*nm*_; 2) $B_{mn}=B_{-m,-n}^{*}$; and 3) $B_{m,-n}=B_{nq}^{*}$, where *q* = *m* − *n*, showing that the information contained in the entire discretized plane (*f*_*n*_, *f*_*m*_) is redundant. Symmetry 1) implies that the bispectral distribution in octants 1 is 2 is symmetric with respect to the first diagonal. Symmetry 2) shows that quadrants 1 and 3 contain equivalent, complex conjugate, information, and the same is true for quadrants 2 and 4. Finally, symmetry 3) shows that octants 1 and 8 contain equivalent information. Therefore, the smallest domain of non-redundant information is octant 1, i.e., the area bounded by the positive frequency axis and the first diagonal (pink in Figure 1). In addition, if the number of frequencies is finite *n* = 1, ⋯ , *N*, as is the case in all numerical applications (e.g., *N* = 12 in Figure 1), the bispectrum is only defined in the area below the second diagonal, because *f*_*m*_ + *f*_*n*_ ≤ *f*_*N*_, hence the triangle appearance of the bispectral plots.

### 2.4. LFP Power

Because the quantity measured by LFP observations is the potential of the electromagnetic field generated by the synaptic pulses, the spectral distribution of LFP variance (i.e., LFP spectral density integrated over some frequency interval) is proportional to the energy of the electromagnetic field per unit volume. Indeed, if the LFP time series *g*(*t*) is a zero-mean, weakly non-linear stochastic process, stationary in the second order statistics (Priestley, 1981; Percival and Walden, 2009), one can show that the discrete Fourier representation has the property

(6) $$\sigma^{2}=\langle g^{2}\rangle =\sum_{n}\frac{1}{2}\langle {\left|a_{n}\right|}^{2}\rangle =\sum_{n}\sigma_{n}^{2},$$

where $\sigma_{n}^{2}=\frac{1}{2}\langle |a_{n}{|}^{2}\rangle =S_{n}^{gg}\Delta f$ is the variance of Fourier mode *n*, *a*_*n*_ is its amplitude, $S_{n}^{gg}$ is the spectral density (Equation 1), and Δ*f* is the frequency band width. The mean energy per unit volume of an electromagnetic wave with the amplitude of the electric potential *g* is *w* = ε*g*^2^, where ε is the dielectric constant of the medium (e.g., Pollack and Stump, 2002; Nolting, 2016). The energy per unit volume of a stochastic electromagnetic field is therefore

(7) $$w=\varepsilon \langle g^{2}\rangle =\sum_{n}\varepsilon \frac{1}{2}\langle {\left|a_{n}\right|}^{2}\rangle =\Delta f\sum_{n}\varepsilon S_{n}^{gg}.$$

In other words, the amplitude squared of the Fourier components of the LFP are proportional to the energy stored in the unit volume by that particular Fourier component. A large number of algorithms have been developed for estimating for second- and higher-order statistics (power spectral density, bispectra, skewness, asymmetry, etc) of such processes. The analysis of the LFP in the current study was based on standard techniques used for variance-stationary signals (Priestley, 1981; Papoulis and Pillai, 2002) as previously described in Sheremet et al. (2016).

### 2.5. Numerical Implementation

All data analysis was performed in Matlab® (MathWorks, Natick, MA, USA) using in-house developed code, as well as code imported from the HOSAtoolbox (Swami et al., 2001) for higher order spectral analysis.

Hippocampal layers were determined from the location of current sources and sinks derived on ripple and theta events, gamma power, and the polarity of the sharp-wave (Buzsáki, 1986; Buzsáki et al., 1986; Bragin et al., 1995; Ylinen et al., 1995; Lubenov and Siapas, 2009; Fernández-Ruiz et al., 2017). Details are given in Zhou et al. 2018.

The rat speed was calculated as the smoothed derivative of position. Raw LFP records sampled at 24 kHz (Tucker-Davis system) were pre-processed by applying a 2-kHz low-pass filter and divided into segments of 2048 time samples (approx. 1 s). Spectra and bispectra were classified by speed by averaging the speed over each of the 1-s LFP segments.

The dynamical and kinetic three-wave equations were integrated using the ODE solvers provided by Matlab®. The implementation is trivial, therefore, to save space, the codes are not provided. The authors will, however, gladly share them upon request.

## 3. Results

### 3.1. Observations: Collective-Action Response to Behavior

**Figure 2:**
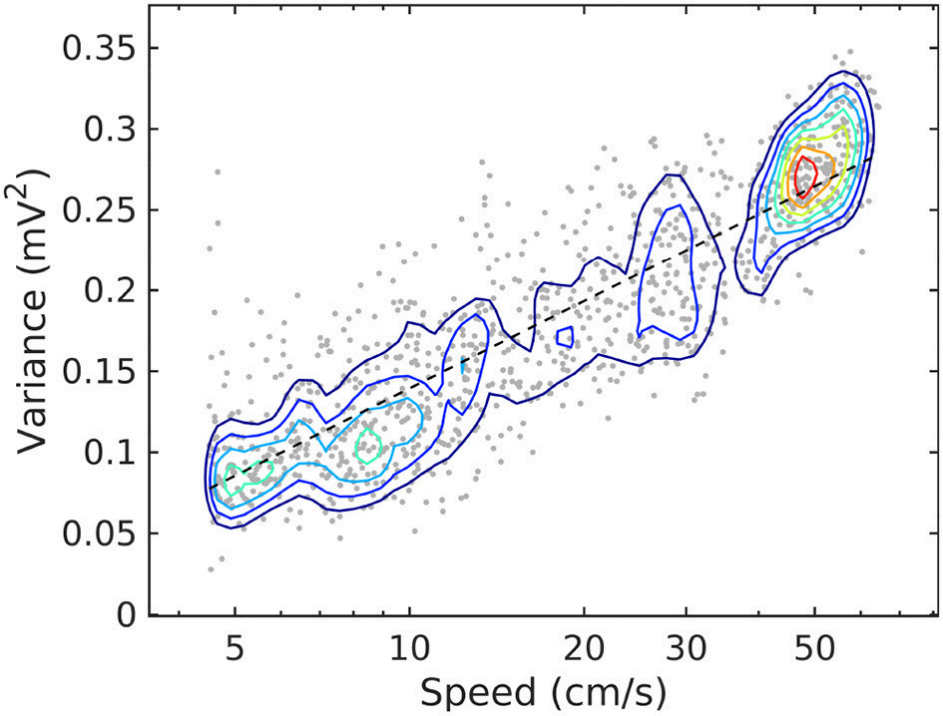
Joint probability density function for LFP variance (LM) and rat speed, for rat r539♂-maurer. The dashed line is a best-fit analytical expression $\left(\frac{v}{1\text{cm/s}}\right)=a\log_{10}\left(\frac{V}{1{\text{ mV}}^{2}}\right)+b$, with *a* = 0.18 and *b* = − 0.04. Dots represent individual realizations (1-s time segments of the LFP recording, section 2). The relation between variance and speed follows the Weber-Fechner law of stimulus perception (Fechner, 1860; Weber, 1860).

The relationship between rat speed and theta and gamma power (Whishaw and Vanderwolf, 1973; Morris and Hagan, 1983; Chen et al., 2011; Ahmed and Mehta, 2012; Kemere et al., 2013; Zheng et al., 2015) is easily observable for speeds roughly above 5 cm/s (e.g., Figure 2). While low speeds, say < 3 cm/s may represent behavior uncorrelated to movement (Zhou et al., 2018), higher speeds exhibit a monotonic correlation with the power of hippocampal activity (firing rates of hippocampal and entorhinal neurons increase with speed; McNaughton et al., 1983; Rivas et al., 1996; Shen et al., 1997; Hirase et al., 1999; Maurer et al., 2005; Kropff et al., 2015). This observation provides a monotonic ordering of LFP statistics, with speed playing the role of an order parameter. Because of the relation is monotonic, the expression “evolution with speed” may be used unambiguously for “change as speed increases” (similar to the standard expression “time evolution” for change as time the time parameter increases). A relation to time exists, since speed itself is fundamentally a function of time: for example, an nominal increase of the speed parameter is in fact associated with time instances of acceleration.

For simplicity, we limit the data used here for illustration to a single representative source, rat r539♂-maurer. A discussion of the consistency of theses trends across the available, small (but growing) population of rats is presented elsewhere (Sheremet et al., 2018; Zhou et al., 2018).

### 3.2. Variance Spectra as a Function of Speed

Here, we examine estimates of spectral density of LFP traces recorded in the str. pyramidale (CA1.pyr), radiatum (CA1.rad), lacunosum moleculare (LM) and upper blade of the dentate gyrus (DG), indexed by rat speed.

**Figure 3:**
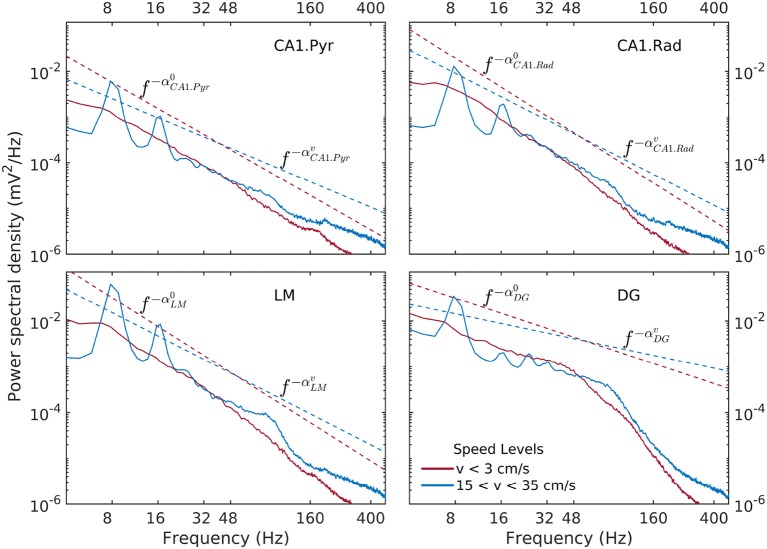
Power spectral density as a function of rat and hippocampal layer for low (red) and high rat speed (blue). The logarithmic representation used in this figure is useful for detecting frequency intervals where the spectrum follows a power law of the form $S\left(f\right)=S\left(f_{0}\right){\left(\frac{f}{f_{0}}\right)}^{-\alpha }$, because in this representation of the the relation *S*(*f*) becomes linear, e.g., $\log_{10}S=\log_{10}S_{0}-\alpha \log_{10}\left(\frac{f}{f_{0}}\right)$. The absolute value α of the exponent is loosely referred to as “spectral slope.” Dashed lines (offset for better visibility) represent power laws shapes *f*^−α^ that approximately match the observed spectra for 6Hz < *f* < 50Hz. Low-speed spectral slopes are $\alpha_{\text{CA1.Pyr}}^{0}\approx 1.9$, $\alpha_{\text{CA1.Rad}}^{0}\approx 2.1$, $\alpha_{\text{LM}}^{0}\approx 2.1$, and $\alpha_{\text{DG}}^{0}\approx 1.1$. High-speed spectral slopes are $\alpha_{\text{CA1.Pyr}}^{v}\approx 1.4$, $\alpha_{\text{CA1.Rad}}^{0}\approx 1.7$, $\alpha_{\text{LM}}^{0}\approx 1.7$, and $\alpha_{\text{DG}}^{0}\approx 0.7$. Data was produced from rat r539♂-maurer.

Spectral densities show a weak, but significant variability as a function of speed and layer (Figure 3). At lowest discernible speeds (*v* ≲ 3 cm/s), all hippocampal layers exhibit non-trivial baseline, lowest-variance spectrum, that can be characterized in general as having a power-law shape *f*^−α^ over the entire range of approximately 6–300 Hz, with the exception of the DG layer, which exhibits a two-slope shape with a break point in the neighborhood of 50 Hz. The absolute value α of the power-law exponent is loosely referred to as “spectral slope”. In the CA1 layers the spectral slope is α ≈ 2 with slight variations (Figure 3). In the DG, the slope of the lower frequency range *f* ≲ 50 Hz visibly smaller, α ≈ 1. At high speeds (*v* > 15 cm/s), the theta rhythm and its harmonics dominate the lower frequency band of the spectrum, and low-power peak appears in the gamma range between 50 Hz and 120 Hz. Overall, the slope of the spectrum decreases. The CA1 layers have similar slopes 1.4 < α < 1.7, with the DG layer again standing out at α ≈ 0.7. The power in the high-frequency tail of the spectrum (*f* > 200 Hz) also increases significantly. In agreement with prior research (Bragin et al., 1995), the CA1.pyr layer shows the least energetic gamma range of all the layers examined.

**Figure 4:**
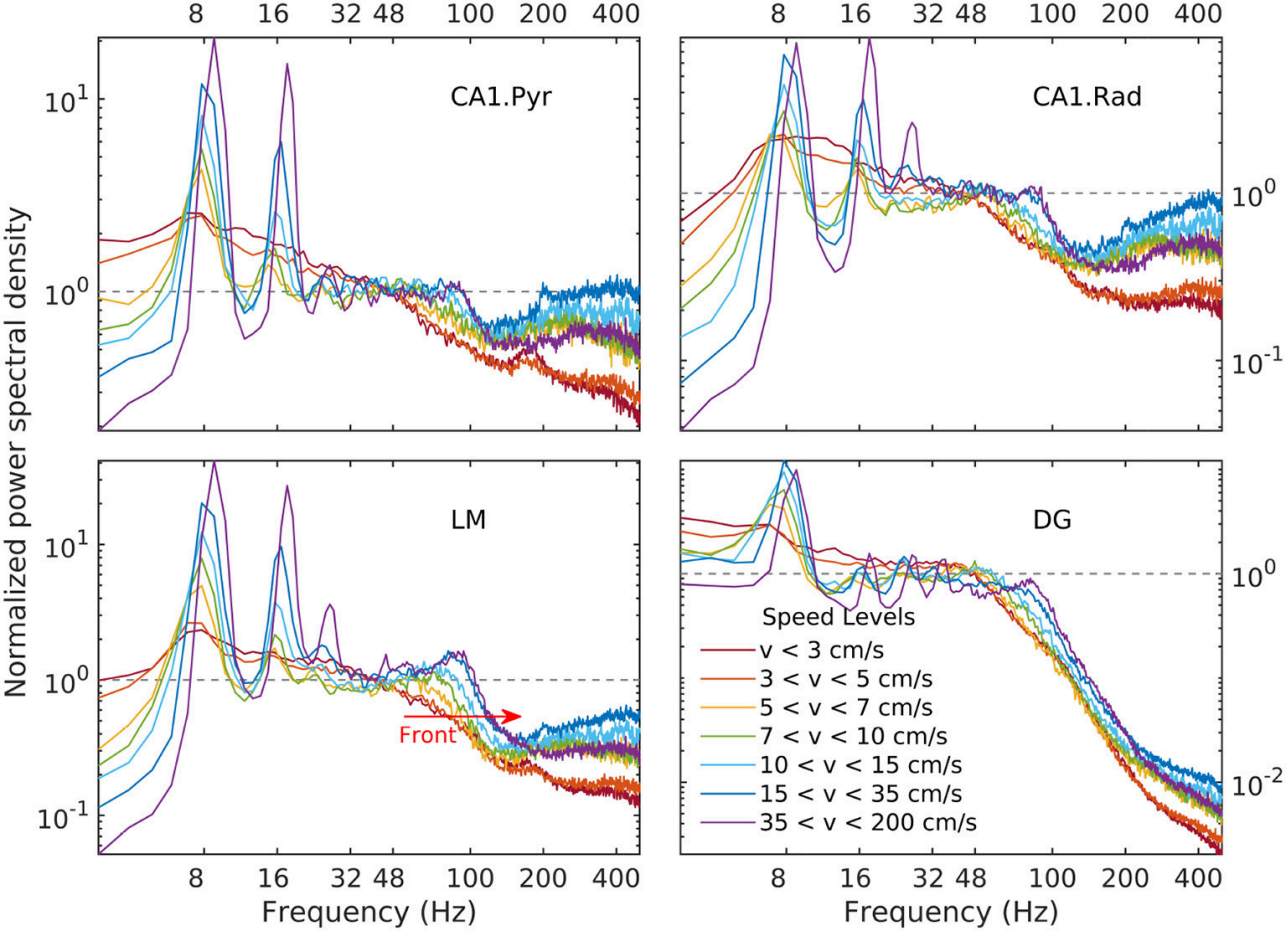
Normalized LFP spectrum estimated for CA1.pyr, CA1.rad, LM, and DG layers as a function of speed. Frequency spectra are normalized by dividing them by the corresponding power law, i.e., $s\left(f\right)=\frac{S\left(f\right)}{S\left(f_{0}\right)}{\left(\frac{f}{f_{0}}\right)}^{\alpha_{\text{layer}}^{v}}$, where *S* is the power spectrum, *s* is the normalized spectrum, and *f*_0_ = 48 Hz. The dependency of the LM gamma band on speed has the appearance of a spectral “front” moving to the right (red arrow), as it steepens and develops a peak. A similar behavior may be seen in all layers. Note that because the limits of vertical axes for the LM and DG layers (lower panels) are different, the spectral front appears to be weaker in the DG layer than in the LM layer. In fact, at the gamma peak (around 80 Hz, lower panels in Figure 3) the spectral densities grow by remarkably similar factors (≈ 3).

Details of the variability of hippocampal LFP spectra with speed are shown in Figure 3 for seven speed intervals. According to Figure 2, speed ordering is statistically equivalent to ordering by total LFP variance. The spectra are normalized by dividing them by *f*^−α^, using the high-speed slopes 1.4, 1.7, 1.7, and 0.7 for CA1. Pyr, CA1.Rad, LM, and DG, respectively. The normalization reduces the spectra to ≈ 1 in the frequency range where it agrees to the power law, and highlights spectral peaks. Although the ordering parameter is total variance, the spectra show a remarkable monotonic ordering in all frequency bands, with the exception of the highest two speed intervals, where the evolution stagnates and perhaps reverses slightly. The normalization re-scaling highlights several features of the evolution as a function of speed. The spectra in all layers tilt as energy increases (e.g., the LM slope changes from 2.1 to 1.7). Theta and its harmonics become dominant in the low-frequency range: four peaks at multiples of 8 Hz may be seen clearly in the CA1.Pyr and DG spectrum, perhaps three in the CA1.Rad and LM spectra. Also remarkable is the peculiar way the gamma range evolves: rather than developing some broad peak in 50–120 Hz range, the gamma range growth seems to result from the *s* ≈ 1 domain (where *s* is the normalized spectrum) progressively extending into the higher frequency range, while a “bump” develops in the neighborhood of *f* ≈ 100 Hz. This evolution is suggestive of a “front” of energy that propagates “against some resistance” toward higher frequency. This type of evolution may be seen in all layers. Although one might expect slight differences between acceleration and deceleration states for the same speed, these are negligible (data not shown). Therefore, the classification by speed does not differentiate between acceleration sign: the transition, say, between 10 cm/s and 20 cm/s may includes both acceleration and deceleration. Thus, the transition process described by Figures 2, 4 (and also below) is reversible, therefore quasi-stationary: all states described by these spectral may be assumed quasi-equilibrium states (in other words, imagining this evolution as a number of time steps, the transition between different steps is slow enough to allow the system to reach equilibrium and every step). This is consistent with the ability of the rat to control its speed. We will return to these ideas below.

### 3.3. Higher-Order Spectra as a Function of Speed

Higher-order spectra provide information about cross-frequency coupling. The bispectrum, the lowest order (and hence, the most “accessible” such estimator) has been used for a long time in wave dynamics (Hasselmann et al., 1963; Rosenblatt and Van Ness, 1965; Coppi et al., 1969). The relationship between its structure and third order statistics of the time series is well-understood (e.g., Haubrich and MacKenzie; Masuda and Kuo; Elgar; for bispectral definitions and terminology, see section 3.3). Although bispectral analysis is not common in neuroscience, recent work (Kovach et al., 2018) has shown that similar, widely used estimators for phase-amplitude and amplitude-amplitude coupling are, in fact, particular implementations of bispectral estimators (containing additional restrictive assumptions that make them susceptible to misinterpretation; e.g., Hyafil, 2015). To save space, we discuss only low (*v* < 10 cm/s) and high (*v* > 35 cm/s) speed levels; intermediate levels (not shown) represent a relatively smooth transition between these two limits. Because of the substantial difference in dynamics between CA1 and DG, we confine our discussion of bispectra to the LM layer.

**Figure 5:**
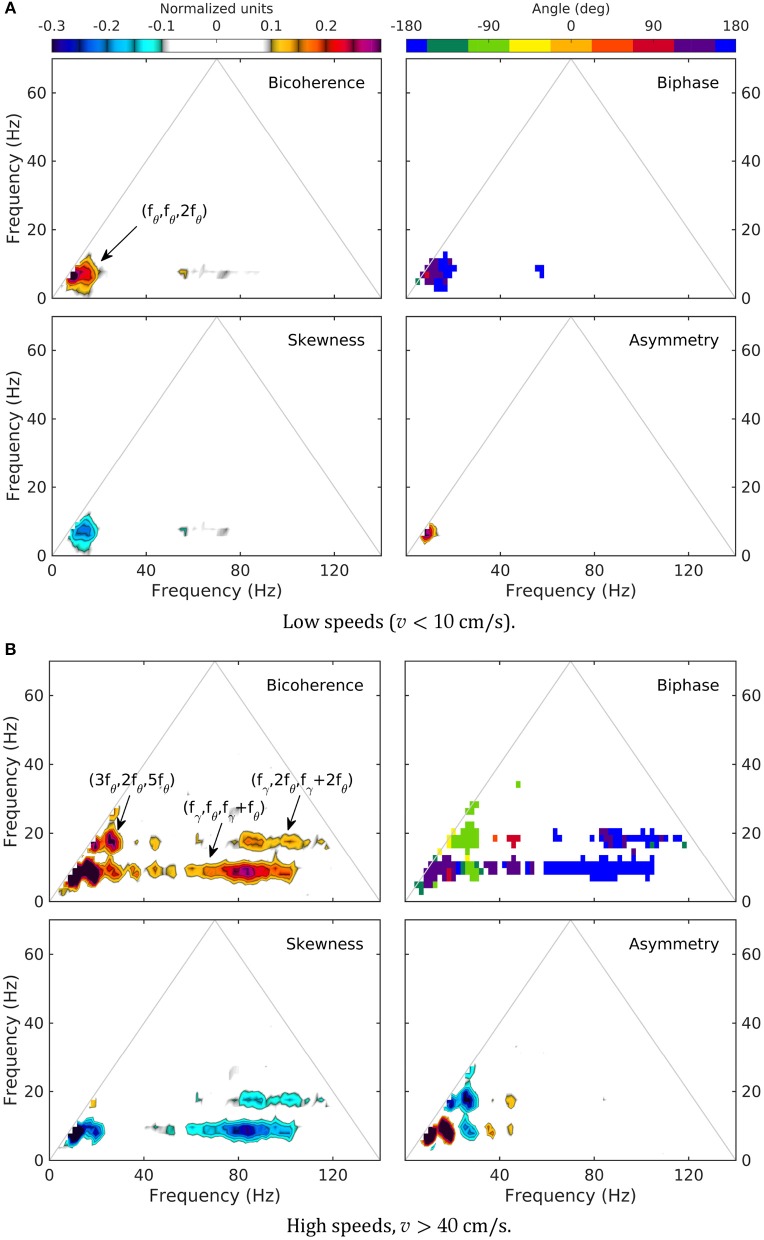
Normalized bispectrum (Equation 5) for the LM layer. The bicoherence is blanked below 0.1 (with 300 DOF, zero-mean bicoherence is < 0.1 at 95% confidence level; Haubrich and MacKenzie, 1965; Elgar and Guza, 1985).

As previously reported (Sheremet et al., 2016), bispectral estimates also show significant variability with rat speed. Low-speed bispectra are statistically zero (Gaussian) overall (Figure 5). The bicoherence exhibits a weak peak at (*f*_θ_, *f*_θ_, 2*f*_θ_), corresponding to the phase coupling between theta (*f*_θ_ ≈ 8 Hz) and a relatively broad band around its 2*f*_θ_ harmonic (see arrow, Figure 5A, bicoherence). The phase of the harmonic band is in part in opposition and part in quadrature with theta (see transition red to blue in Figure 5, biphase), thus contributing overall to negative skewness of the LFP (peak is negative in 5a, skewness), but positive asymmetry (5a, asymmetry; see definitions in section).

In contrast, high speed activity show rich phase-coupling structures, involving theta and gamma (Figure 5B). One may separate two frequency regions corresponding to the coupling between theta and its harmonics, and theta and gamma. The picture of the coupling between theta and its harmonics agrees with the spectral evolution (Figure 3) and previously reported results (Sheremet et al., 2016). The bicoherence exhibits significant levels of phase coupling, reaching as high as (5*f*_θ_, *f*_θ_, 6*f*_θ_), (3*f*_θ_, 2*f*_θ_, 5*f*_θ_), and (3*f*_θ_, 3*f*_θ_, 6*f*_θ_). The relationship between harmonics and theta is quite diverse, with some harmonics contributing to LFP skewness, and others only to LFP asymmetry. For example (Figure 5B, skewness and asymmetry), the coupling (*f*_θ_, *f*_θ_, 2*f*_θ_) generates both negative skewness and positive asymmetry, while (2*f*_θ_, 2*f*_θ_, 4*f*_θ_) results in negative asymmetry only. Theta-gamma coupling is prominent at high speed, engaging theta harmonics, and contributing strongly to negative skewness (Figure 5B).

### 3.4. Summary of Observations

**Figure 6:**
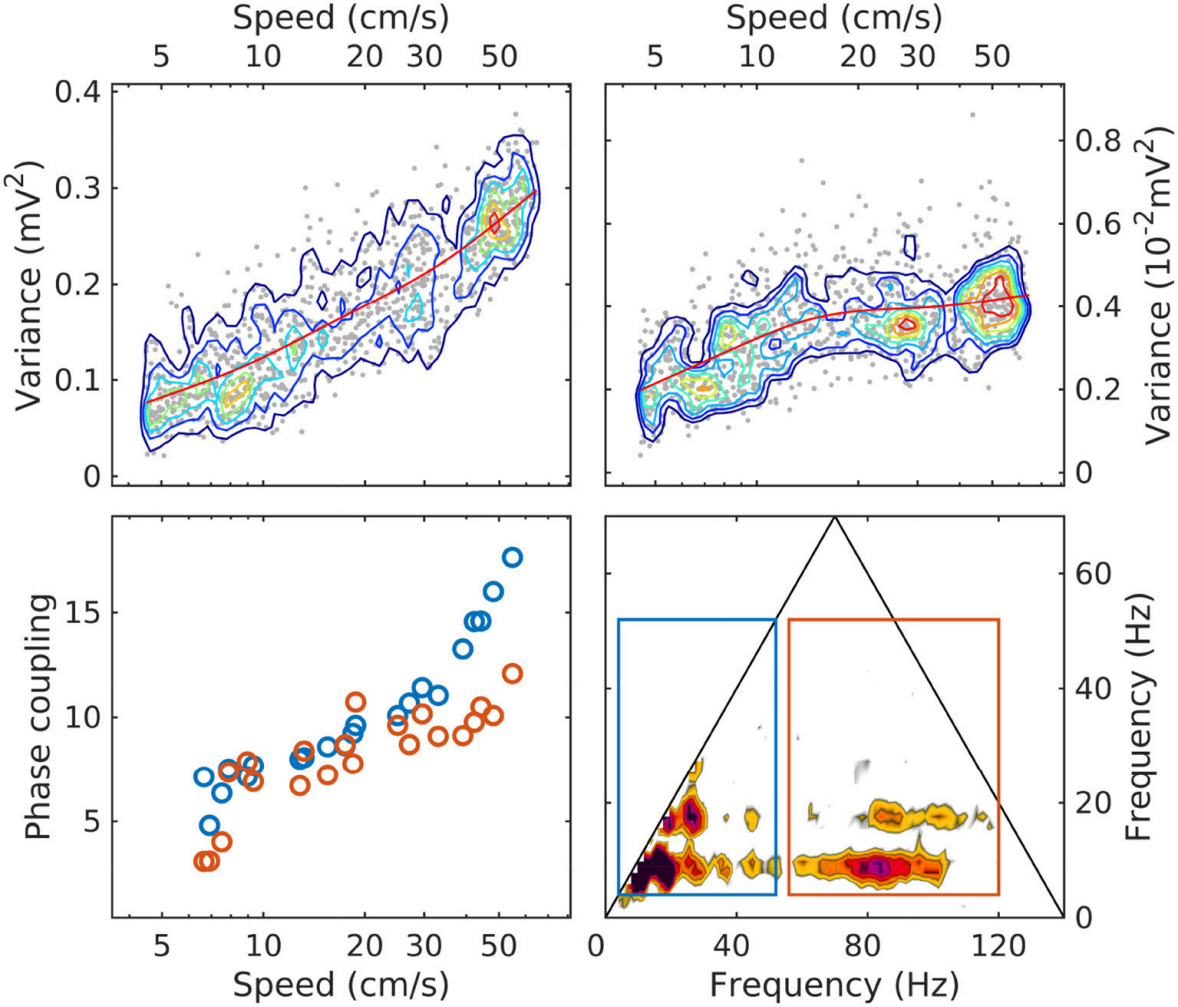
The evolution with speed of LM theta power (upper left); gamma power (upper right; note the different units); phase coupling of theta and its harmonics (lower left, blue); phase coupling of theta and gamma (lower right, red). The phase coupling measure is the bicoherence integrated over the area of the rectangles shown in the lower-right panel (same colors as in lower-left panel). Because the bicoherence is normalized, the units of the phase coupling measure are arbitrary. In upper panels, the red line is a moving average, included to highlight the evolution trend.

Our observations show that sorting LFP epochs by speed provides an efficient classification device that produces remarkably well-ordered spectra and bispectra. As summarized in Figure 6, with increased speed the total LFP power increases, as well as the power in the theta and gamma bands. The power in the theta band grows overall by a factor of 4 at a relatively steady rate as a function of speed, while gamma grows by about a factor of 2 and seems to plateau. Phase coupling (as measured by the bicoherence integrated over the theta/harmonics and theta/gamma frequency domains) shows a steady growth as a function of speed, accelerating at high speeds. Spectral slopes (α) decrease, consistent with an accumulation of energy in the gamma range; and phase coupling involving theta and gamma increases.

The process of evolution with speed is reversible. Recall that we refer to evolution as change with increased speed, therefore the “reverse” process is the transformation corresponding to decreasing speed. The reverse process appears to converge as *v* → 0 (*v* is speed) toward a limiting, “background” state. Ignoring for now other forms of activity not taken into account here, the background state represents “inactive” behavior, whereas the active state would be associated with significant speed levels. This hypothetical background has some remarkable properties. Although its spectrum decays faster than any active-state spectrum (higher slope α), it still contains significant power (in Figure 2, about $\frac{1}{4}$ of the most active state). In addition, the LFP is nearly Gaussian, i.e., exhibits overall no phase coupling (ignoring the weak theta signal).

The evolution with speed of hippocampal power distribution over scales is strongly suggestive of cross-scale energy exchanges, statistically directed from low to high frequencies (from large to small scales). Cross-scale energy transfers can only result from non-linear interaction between scales; however, there is no obvious reason for a statistically preferential direction of transfer, unless the physical system is perturbed in a way that forces a certain internal exchange. The apparent direction of the cross-scale energy flow is consistent with the system receiving energy at the large scales (low frequency end) and losing energy at the small scales (high frequency end). The collapse, in the range *f* < 12 Hz, of the background spectrum as theta itself progressively concentrates all spectral power suggests that the background spectrum is not associated with the energy input and represents a completely different process, replaced at large scale by theta during task behavior. This shift and the considerable increase in theta power are consistent with theta playing an important part in the energy input process (please see next section for further discussion).

In our observations, the evolution of the scale distribution of LFP power and non-linearity exhibits some obvious, yet unexplained features. Why do spectral slopes change? Why does phase coupling develop? Why does theta develop harmonics, but gamma does not, coupling instead to theta? How is the collective action energy balance related to the background spectrum?

### 3.5. The Weak Turbulence Framework

**Figure 7:**
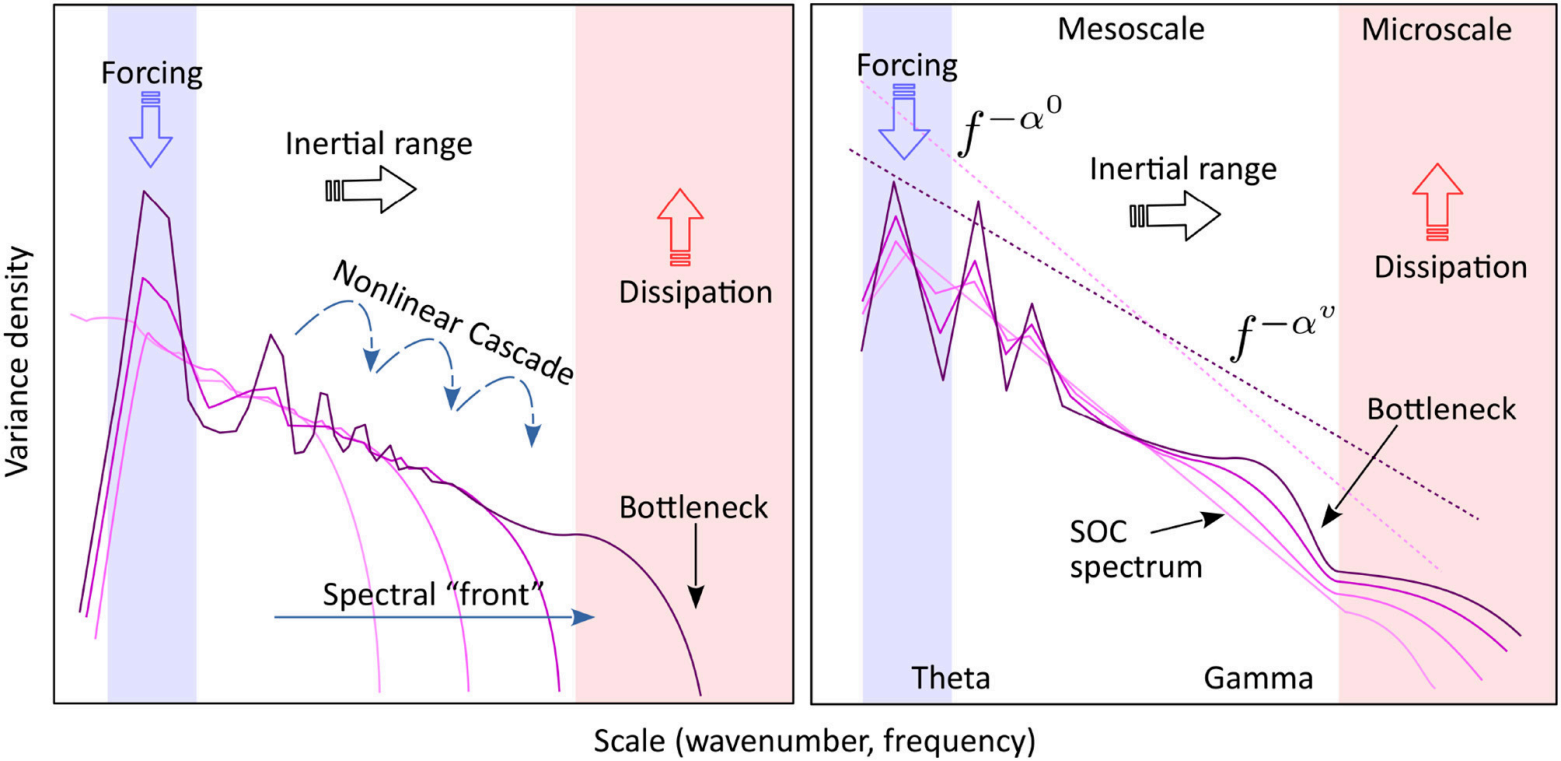
**Left:** The turbulence model. Energy is introduced into the system at the forcing scale (blue) is separated from the dissipation scale (red) by a “transparent” inertial range (white), largely free of forcing and dissipation. Non-linear interactions generate a cross-scale energy flux (cascade) from the source to the sink. Gray curves show the possible evolution of the spectrum toward stationary state, if initially the inertial range contains no energy. The stationary spectrum (purple) corresponds to a constant spectral flux of energy across the inertial range. A “bottleneck” in the spectral flux capacity at small scales may cause an accumulation of energy (spectral bump) at larger nearby scales. The axes are in logarithmic scale. **Right:** A schematic of the observed spectral evolution, interpreted in a way similar to turbulence. The light-colored spectrum is the background, corresponding to low activity (speed), and representing the self-organized critical (SOC) state. Increasingly dark lines represent the spectral shape at increasing intensity of activity (speed). The low-frequency peaks represent the theta rhythm and its harmonics (marked collectively as “theta”). A feature similar to the turbulent spectral front is observed in the gamma range. A spectral bump similar to a bottleneck (e.g., L'vov et al., 2007; Meyers and Meneveau, 2008; Proment et al., 2009; Nazarenko, 2011, see note in the text) is observed in the high-frequency gamma range.

Figure 7 compares side by side the standard conceptual model of turbulence with a schematic of observed hippocampal LFP evolution with energy input (speed).

The main focus of the turbulence theory is the internal energy^7^ balance in a non-linear, multi-scale physical system whose scales interact and exchange energy (Figure 7, left). If the energy source and sink of the system are well-separated in scale, for example, the source is located at large scales, and the sink at small scales, there exists an intermediate domain, called inertial range, where the only energy process is the cross-scale energy flow. Assume that the input rate at the source is constant, and the system has initially zero energy. If the scale interaction is local (i.e., the strongest interaction is between similar scales) input energy will accumulate initially in a band of scales adjacent to the source. As the energy in that band increases, local non-linear exchanges intensify (non-linearity increases with energy), and the energy introduced into the system flows downscale. Eventually, the energy flow (non-linear cascade) reaches the dissipation (sink) scales, where it is removed from the system. The stationary state will occur when the dissipation rate matches the input rate, and will be characterized by a constant flux of energy across the inertial range, from source to sink^8^. The cross-scale energy flux is turbulent cascade (Richardson, 1922). One of the most celebrated results of hydrodynamic turbulence is the Kolmogorov's (1941) argument that stationary spectrum follows the power law *k*^−5/3^, where scales are represented by the wavenumber *k*.

But for the presence of a background spectrum, the similarity between the standard turbulence model and LFP spectral evolution are striking. The analogy suggests that theta is the source of energy for collective action, and microscopic processes are as the main energy sink, while the mesoscale acts largely as an inertial window that allows for a cross-scale energy flow from source to sink. The development of the gamma peak, similar to the bottleneck effect in hydrodynamics, may be interpreted as the existence of a transitional scale right above microscopic, that has a limited energy-flux ability, thus causing an accumulation of energy at intermediate scales. The observation that spectral evolution with speed is reversible (in other words, that spectra at each speed represent an equilibrium state) is also remarkable, because it is consistent with Kolmogorov stationary spectra of turbulence.

#### 3.5.1. Mesoscopic Turbulence on an Active Network

Therefore, turbulence framework could be useful for collective action dynamics, *provided that* the mesoscale may be identified with the inertial range; or, equivalently, if non-linearity dominates dissipation at mesoscale. However, while observations of propagating theta waves suggest that collective action is weakly dissipative, this seems to contradict the well-known strongly-dissipative character of microscopic neuronal dynamics^9^. Furthermore, the presence and role of the background spectrum deserves some discussion.

From the perspective of collective action, the hippocampus behaves as an “active” network, i.e., a physical system of active elements that contain a certain amount of energy which they release in explosive bursts when activated by a threshold type of trigger. The statistical state of the network may be described by a probability distribution of internal energy around a mean level, sustained by energy (pulses) channeled through network connections. Even if the mean internal energy level is below the threshold, a small number of elements will have energy exceeding the threshold, and will fire. A fraction of the burst energy is recaptured and redistributed through the network connections to maintain a mean level of internal energy. After bursts, network elements go through a “recharge” (refractory) period. Note that, in the absence of external input, bursts are the only energy source for maintaining the internal energy level. If no bursts occur, the system collapses. In an equilibrium state, the probability distribution of the internal energy should have a standard deviation large enough to maintain the mean.

If the state described above is perturbed by adding energy over a mesoscopic area, the internal energy distribution shifts toward the threshold, increasing the number of bursts. The perturbation decays or grows in time depending on whether the ratio of energy recaptured from bursts to the energy of the initial perturbation smaller or larger than 1. If this fraction is ≈ 1, the perturbation is self sustained.

*We conjecture that self-sustained collective action is the behaviorally meaningful hippocampal activity*. Thus, the relevant type of collective action is only weakly dissipative. Remarkably, it also likely requires a non-zero background activity (background spectrum, section 3.1). Indeed, for a self sustained perturbation, both the energy recaptured from bursts and the energy of the perturbation depend on the mean energy level of the system. A likely mechanism to maintain an equilibrium level of internal energy, and its required standard deviation, is to randomly trigger multi-scale burst patterns. This idea circles back to the concept of self-organized criticality (SOC) (SOC; Bak et al., 1988; Beggs and Plenz, 2003; Beggs and Timma, 2012; Pruessner, 2012) and metastability (Tognoli and Kelso, 2014). The featureless, power-law, Gaussian background spectrum we observe (Figure 3) is consistent with the “edge of chaos”, self-organized critical background state for optimal transmission of collective action. In this context, SOC becomes an important element of brain dynamics.

The relation between background SOC state (rest) and collective-action turbulence (intense task behavior) might also explain the behavior of the LFP power spectra in the low frequency range *f* < 12 Hz (see discussion at the end of section 3.1). If the excess energy for sustaining the collective action is provided (as observations suggest) by increasing theta power, much of the large scale activity is taken over by organized theta action, thus restricting the large scale extent of SOC spectra.

#### 3.5.2. The Weak Turbulence Model

The turbulence framework may now be formalized. The turbulence theory investigates the internal balance of a multi-scale system. It studies the interaction efficiency as a function of scale (e.g., resonance conditions); the development of cross-scale coupling correlations between scales; characteristics of the long term evolution of the system; the existence of equilibrium spectra associated with cascades of conserved quantities (e.g., power law spectra that maintain a non-zero cross-scale energy flux); non-stationary evolution patterns (e.g., bottleneck formation); and other characteristics of the non-linear, cross-scale exchange mechanisms related to conservation laws.

These are precisely the features that characterized the evolution with speed of our LFP observations (e.g., Figure 4).

Here we give only the a brief elementary discussion of the basic equations that govern weak turbulence. The principles of the weak turbulence model, some derivation algebra, and some results are further discussed in the Supplementary Material. We caution, however, that presentation is an oversimplified, retold version, and that the full theory is much richer and complex. We encourage the interested reader to consult the original sources, written by the fathers of the theory: the comprehensive monograph (Zakharov et al., 1992), excellent review papers by Zakharov (1999), Newell et al. (2001), and Newell and Rumpf (2010), and the account by Nazarenko (2011), that includes more recent results.

#### 3.5.3. Dynamical Equation

The focus of the turbulence theory is to describe the internal energy balance of a multi-scale system, i.e., the evolution of the distribution of energy over scales (the spectrum), the cross-scale energy flux (the energy cascade), associated non-linear cross-scale coupling, and other quantities. The general way to achieve this is to define the state of the system using a “state variable”, for example internal energy, and write its evolution using conservation laws. For example, in thermodynamics the rate of change of internal energy is equal to the heat and mechanical work exchanged by the system with the environment.

For a spatially-distributed system such as collective action, the state variable becomes a function of space and time (a field), and the evolution equation becomes a partial differential equation. The exact form of these equations for collective action will be presented elsewhere. Here, it suffices to note that the internal energy *field* for hippocampal collective action may be defined proportional to the mean neuron potential per unit area, and the conservation of the internal energy involves a balance of electric pulses sent down the network connection and the energy recaptured by the network from bursts. This approach leads to equations similar to the Wilson and Cowan (1972, 1973); Cowan et al. (2016) model, although its form and derivation methodology is different and somewhat less consistent.

To avoid having to reference the specifics of the system, *for illustration purposes only* we will tentatively identify the internal energy with the Hamiltonian, which will conveniently allow us to introduce the equations of weak turbulence in a general form, without having to reference the specifics of the system. The Fourier representation of the collective-action field provides a scale decomposition of the internal energy field, so in principle, the Fourier transform of the conservation law (Hamilton's equations) yields an equation for the Fourier amplitude *a*(*k, t*) of the collective action component with wave number *k*. The general form of this equation is:

(8) $$i{\overset{•}{a}}_{{}_{k}}=\omega_{k}a_{k}+\frac{1}{2}\iint_{-\infty }^{\infty }\left(V_{k;12}a_{1}a_{2}\delta_{k;12}^{k}+2V_{1;k2}^{*}a_{1}a_{2}^{*}\delta_{1;k2}^{k}\right)dk_{12}+\ldots$$

where Fourier collective-action mode (or simply, mode) is identified by its wavenumber *k* and frequency *f*(*k*), (we assume that the dispersion relation has a single root). In Equation (8), the notations are: ω(*k*) = 2 π *f*(*k*) is the radian frequency; $\overset{∙}{a}=\frac{da}{dt}$; *V*_*k*; 12_ = *V*(*k*; *k*_1_, *k*_2_) is the interaction coefficient; and δ is the Dirac delta function. We use the shorthand notation σ_1,2_ = σ (*k*_1,2_), σ_*k*_ = σ (*k*), and $\delta_{k;12}^{\sigma }=\delta \left(\sigma_{k}-\sigma_{1}-\sigma_{2}\right)$, where σ is some quantity depending on *k*; and also *dk*_12_ = *dk*_1_*dk*_2_. For simplicity, we limit the discussion quadratic non-linearity (right-hand side terms of the form *a*^2^) terms, but a full description might require including cubic (*a*^3^), and possibly higher-order non-linearity (e.g., the non-linear Schrodinger equation, Newell, 1985; or the Ginzburg-Landau equation Ermentrout, 1981; Cross and Hohenberg, 1993; Passot and Newell, 1994; Ermentrout et al., 1997).

Two equations for the modulus *b* ≥ 0 and phase θ of *a*(*k, t*) are obtain substituting *a*(*k, t*) = *b*(*k, t*)*e*^*iθ*(*k, t*)^ into Equation 8

(9a) $$\begin{array}{l} {\overset{•}{b}}_{k}=-\frac{1}{2}\iint_{-\infty }^{\infty }V_{k;12}b_{1}b_{2}\sin\Delta_{k;12}^{\theta }\delta_{k;12}^{k}\\ \;+\frac{1}{2}\iint_{-\infty }^{\infty }2V_{1;k2}^{*}b_{1}b_{2}^{*}\sin\Delta_{1;k2}^{\theta }\delta_{1;k2}^{k}dk_{12}, \end{array}$$

(9b) $$\begin{array}{l} {\overset{•}{\theta }}_{k}=-\omega_{k}+\frac{1}{2}\iint_{-\infty }^{\infty }V_{k;12}\frac{b_{1}b_{2}}{b_{k}}\cos\Delta_{k;12}^{\theta }\delta_{k;12}^{k}\\ \;+\frac{1}{2}\iint_{-\infty }^{\infty }2V_{1;k2}^{*}\frac{b_{1}b_{2}^{*}}{b_{k}}\cos\Delta_{1;k2}^{\theta }\delta_{1;k2}^{k}dk_{12}, \end{array}$$

where we used the notation $\Delta_{k;23}^{\theta }=\theta_{k}-\theta_{1}-\theta_{2}$.

*Meaning of the dynamical Equation 8*.

*(i)* Temporal (frequencies) and spatial scales (wavenumbers) are related through the dispersion relation (see the Supplementary Material), therefore only one of the parameters *k* and *f* is independent. The choice of the independent parameter is arbitrary, because the dispersion relation is invertible. The *f*(*k*) representation resolves the spatial structures and yields a time-evolution equation. The *k*(*f*) representation resolves the time structure and is appropriate for time-series analysis (e.g., LFP recordings). Therefore, equations are written here in *f*(*k*), but observational data is discussed in the *k*(*f*) representation. Below, the concepts of “frequency”, “wavenumber”, “mode” and “scale” will be treated as equivalent and interchangeable.

*(ii)* Equation 8 is called dynamical equation. If the Hamiltonian is identified with energy, the quantity |*a*|^2^ has the physical dimensions of action (energy × time). The form of Equation (8) in universal in the sense that the details of the physics of the system are contained the coefficients ω_*k*_ and *V*_*k*; 12_ only.

*(ii)* Equation (8) describes the non-linear interaction of mode *k* with the pair of modes (*k*_1_, *k*_2_). A triplet of interacting modes (*k, k*_1_, *k*_2_) is called a “triad”. The strength of the interaction depends on the interaction coefficient *V*_*k*; 12_. The factor $\delta_{k;12}^{k}$, resulting from the orthogonality of the Fourier representation, is a selection criterion: interacting modes satisfy the equation

(10) $$\Delta_{k;12}^{k}=k-k_{1}-k_{2}=0.$$

**Figure 8:**
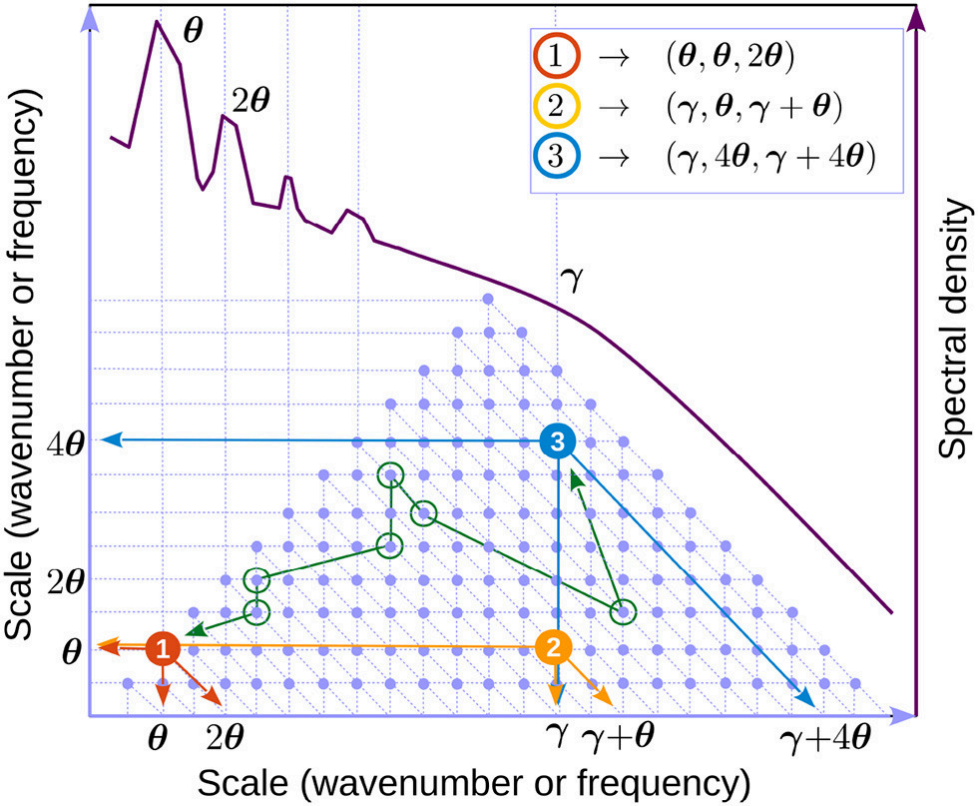
Discrete representation of interacting triads (Equation 8). The underlying grid geometry is the same as for the bispectral representation (see section 2, and also Figure 1). Because *k* and *f* are interchangeable, we refer to either as “scale”. Light blue dots represent triads. The axes represent scale 1 and 2 in the triad (e.g., *k*_1_ and *k*_2_ in Equation 10). The third interacting mode (*k* in Equation 10) may be found by as the intersection of the second diagonal passing through the triad with the horizontal axis (e.g., arrows in triad 1, red dot). One can easily check that the triangle of blue dots represents all possible triads for the scale interval shown (hence the triangular shape of the bispectrum). A schematic of a LFP spectral shape (dark red) is used to indicate possible locations for the theta (θ) and gamma (γ). Example of triads involving theta and gamma are identified by colored dots (compare with the annotations in Figure 5B). Green circles mark an example (arbitrarily-chosen) of a chain of triads connecting triads 1 and 3. The order of connection is marked with a green line. One can check that each pair of consecutive circles share one mode. All triads in the spectrum are connected by many such chains.

It is useful to think of Equation (8) in a discretized form, e.g., replacing the integrals by sums. A schematic representation is shown in Figure 8.

*(iv)* Equations (9) show that non-linear interaction result in both amplitude and phase evolution. The effectiveness of non-linearity depends on modal amplitudes, on the interaction coefficient, and (importantly) on the phase mismatch $\Delta_{k;12}^{\theta }$. If $\Delta_{k;12}^{\theta }$ is large, the non-linear term oscillates fast and the effect small; if $\Delta_{k;12}^{\theta }$ is small, the non-linear term preserves sign over longer periods of time and the effect is significant. If $\Delta_{k;12}^{\theta }$=0, the contribution is maximal.

*(v)* The non-linear contributions to modal phase evolution (Equation 9b) have same properties as those in the amplitude Equation 9a, with the important difference of *b*_*k*_ appearing at the denominator. If *b*_*k*_ → 0 (mode *k* has small amplitude) the non-linear phase component becomes arbitrarily large and dominates the total phase. Therefore, the phase of low-amplitude waves is “dictated” by non-linear forcing.

*(vi)* The dynamical equation 8 is deterministic: may be integrated exactly to obtain the state of the system at time *t* if the initial value of amplitudes *a*(*k, t*_0_) are known (*t* > *t*_0_). To understand the general energy balance (e.g., spectral evolution), however, one is not interested in particular realizations, but in the predictions the equation provides for averaged quantities such as the spectrum or bispectrum. In practice, the averaging operator 〈·〉 used in Equations (1–2) may be replaced by averaging over initial phases (see the discussion of random phase average in Nazarenko 2011). Therefore, estimates of spectra and bispectra may be obtained by integrating Equation (8) many times with different sets of initial phases and, for example for the spectrum, averaging $|a_{k}{|}^{2}$ over all integrations.

#### 3.5.4. The Kinetic Equation

The last remark above suggests averaging the equation itself, rather than averaging the solutions. Briefly (see Supplementary Material), averaging introduces a hierarchy of averages of amplitude products (correlators), such as $\langle a_{1}^{*}a_{2}\rangle$, $\langle a_{1}^{*}a_{2}a_{3}\rangle$, $\langle a_{1}^{*}a_{2}^{*}a_{3}a_{4}\rangle$, and so on, where the angular brackets denote the ensemble average. Assuming spatial homogeneity implies that

(11) $$\langle a_{1}^{*}a_{2}\rangle =n(k_{1})\delta (k_{1}-k_{2})=n_{1}\delta_{1;2}^{k},$$

(12) $$\langle a_{k}^{*}a_{1}a_{2}\rangle =B_{k;12}\delta (k-k_{1}-k_{2})=B_{k;12}\delta_{k;12}^{k}.$$

The quantity *n*(*k*) represents the action density, or “occupancy number”, or “number of particles” (by analogy with quantum mechanics). In statistics *n* and B are called “spectrum” and “bispectrum”, respectively. The quasi-Gaussian assumption results in a system of two coupled equations for the spectrum and bispectrum

(13a) $$\frac{dn_{k}}{dt}=\iint_{-\infty }^{\infty }\left(ℑ\left\{V_{k;12}B_{k;12}\right\}+2ℑ\left\{V_{1;k2}^{*}B_{1;k2}^{*}\right\}\right)dk_{12},$$

(13b) $$\left(i\frac{d}{dt}+\Delta_{k;12}^{\omega }\right)B_{k;12}=-V_{k;12}^{*}\delta_{k;12}^{k}n_{k}n_{1}n_{2}\left(\frac{1}{n_{k}}-\frac{1}{n_{2}}-\frac{1}{n_{2}}\right),$$

Assuming additional long-time regularity conditions reduces the system to single equation called the *kinetic* equation

(14) $$n_{k}=\pi \left(R_{k;12}-R_{1;k2}-R_{2;k1}\right),$$

(15) $$R_{k;12}=\iint_{-\infty }^{\infty }{\left|V_{k;12}\right|}^{2}n_{k}n_{1}n_{2}\left(\frac{1}{n_{k}}-\frac{1}{n_{2}}-\frac{1}{n_{2}}\right)\delta_{k;12}^{k}\delta_{k;12}^{\omega }dk_{12},$$

where $\delta_{k;12}^{\omega }=\delta \left(\Delta_{k;12}^{\omega }\right)=\delta \left(\omega_{k}-\omega_{1}-\omega_{2}\right)$.

*Meaning of kinetic Equation 14*.

*(i)* Equation (13a) highlights the dynamical significance of the bispectrum as the non-linear forcing in the evolution of the spectrum. If the bispectrum cancels, non-linear interactions cancel and the system is effectively, *on average*, linear.

*(ii)* The kinetic Equation (14) or the more general system of Equations 13, represent a stochastic, ensemble-averaged description of the system 8. Kinetic equations of the type of Equation 14 were introduced in statistical mechanics by Boltzmann (e.g., Boltzmann, 1872, 2003; Alexeev, 2004), and are powerful tools in the study of multiple-scale system.

*(iii)* Equation (14) states that in the long-time limit the only interactions that are effective are due to triads that satisfy the *resonance* conditions imposed by the factors $\delta_{k;12}^{\omega }\delta_{k;12}^{k}$, i.e., satisfying the conditions,

(16a) $$\Delta_{k;12}^{k}=k-k_{1}-k_{2}=0,$$

(16b) $$\Delta_{k;12}^{\omega }=\omega (k)-\omega (k_{1})-\omega (k_{2})=0,$$

equivalent to the “maximal” effectiveness of non-linear interaction (see discussion of Equations 9). Whether or not Equation (8) has resonant triads depends on the linear properties of the physical system. The resonance conditions play an important role in the stochastic theory (e.g., Zakharov et al., 1992; Nazarenko, 2011; Anenkov and Shrira, 2018).

#### 3.5.5. Stationary Spectra

Stationary spectra ($\overset{∙}{n}\left(k\right)=0$) are of importance for systems whose evolution is a quasi-equilibrium process. Equation (14) has two classes of stationary solutions (e.g., Nazarenko, 2011).

The Rayleigh-Jeans (RJ) class of spectra comprises the stationary solutions *k*^−1^, ω^−1^, and ${\left(c_{\omega }\omega +c_{k}k\right)}^{-1}$, that obviously cancel the integrand in Equation (14), and correspond to zero exchange across scales. Therefore RJ spectra correspond to thermodynamic equilibrium and equipartition of momentum (*nk*) and energy (*nω*). Realistic, non-isolated systems do not typically reach thermodynamic equilibrium, therefore RJ spectra are not important for the weak turbulence framework, which includes sources and sinks as essential elements.

The Kolmogorov-Zakharov (KZ) spectra are different class of stationary spectra, that correspond to a constant spectral flux ∂_*k*_*F*_*q*_ = 0, *F*_*q*_(*k*) ≠ 0 across the inertial range. They were derived for Equation (14) by Zakharov and Filonenko (1967a,b). They are important for systems in which the dissipation sink can absorb arbitrary rates of energy. Remarkably, they are realized as a non-trivial power law spectrum *n*^KZ^ ∝ *k*^ν^, with ν < 0, ν ≠ − 1. The slope ν of the spectrum is a value that reflects the dimensionality of the system, as well as its linear non-linear properties (homogeneity degrees of the interaction coefficient and dispersion relation).

### 3.6. A Demonstration: Dynamics and Kinetics of a Single Triad

Some features of the high-speed bispectra are consistent with stationary solutions of the dynamical equations 8. We illustrate this using a simplified, universal toy model derived from the framework dynamical equation by considering a single triad of modes *k* = κ_1_, *k*_1_ = κ_2_, *k*_2_ = κ_3_, with κ_1_ + κ_2_ = κ_3_ (see Equation 10). If interactions with all other modes are ignored (equivalent to a single blue dot in Figure 8), Equations (8) to reduce to three equations, called the “three-wave system” (see Supplementary Material; Craick 1985; Rabinovich and Trubetskov 1989). Written in amplitude/phase these are

(17) $$\begin{array}{l} \;{\overset{•}{b}}_{1}=2V_{3;12}b_{2}b_{3}\sin\Delta_{3;12}^{\theta };\;\overset{•}{b_{2}}=2V_{3;12}b_{1}b_{3}\sin\Delta_{3;12}^{\theta };\\ \;\overset{•}{b_{3}}=-2V_{3;12}b_{1}b_{2}\sin\Delta_{3;12}^{\theta },\\ {\left(\Delta_{3;12}^{\theta }\right)}^{•}=-\Delta_{3;12}^{\omega }-2V_{3;12}\left(\frac{b_{1}b_{2}}{b_{3}}-\frac{b_{2}b_{3}}{b_{1}}-\frac{b_{1}b_{3}}{b_{2}}\right)\cos\Delta_{3;12}^{\theta }{}^{\prime } \end{array}$$

where *b*_*j*_ and θ_*j*_ are amplitudes of modes κ_*j*_, *j* = 1, 2, 3, and $\Delta_{3;12}^{\theta }=\theta_{3}-\theta_{1}-\theta_{2}$. Averaged over realizations, the quantity $\Delta_{3;12}^{\theta }=\theta_{3}-\theta_{1}-\theta_{2}$ may be identified with the biphase (see section 3.3). If the triad is resonant, the kinetic version of the three wave equation is (e.g., Rabinovich and Trubetskov, 1989)

(18) $$\overset{•}{n_{1}}=\overset{•}{n_{2}}=-\overset{•}{n_{3}}=2\pi {\left|V_{3;12}\right|}^{2}n_{1}n_{2}n_{3}\left(\frac{1}{n_{3}}-\frac{1}{n_{1}}-\frac{1}{n_{2}}\right).$$

A large body of literature is available that investigates the relevance and dynamics of single-triad interactions in many physical situations, including plasma physics (Coppi et al., 1969; Weiland and Wilhelmsson, 1977; Craick, 1985), non-linear optics (Ablowitz and Segur, 1981; Boyd, 2003), internal oceanic waves (Phillips, 1977; Craick, 1985), and other fields.

One may readily check that a stationary solution of Equations (17) is given by the conditions: $\Delta_{3;12}^{\Theta }=0$ or $\Delta_{3;12}^{\Theta }=\pi$; $\Delta_{3;12}^{\omega }=0$ (the triad is resonant, Equations 16), and that

(19) $$\frac{1}{b_{3}^{2}}-\frac{1}{b_{1}^{2}}-\frac{1}{b_{2}^{2}}=0.$$

The last equation has, for example, the trivial solution $b_{j}^{2}={\left(c_{\omega }\omega +c_{k}k\right)}^{-1}$(RJ spectrum, thermodynamic equilibrium). In other words, stationary state solutions of the framework equations exhibit naturally 8 a biphase of 0 or π.

Applied to the triad formed by theta and its first harmonic (θ, θ, 2θ), i.e., κ_1_ = *k*_θ_, κ_2_ = *k*_θ_, and κ_3_ = *k*_2θ_ (red dot in Figure 8) this result is consistent with observations that show theta and in first harmonic are in phase (see Figure 5B, upper-right panel). The effect of this type of coupling is to sharpen the crests and flatten the troughs of the time series, generating positive skewness.

**Figure 9:**
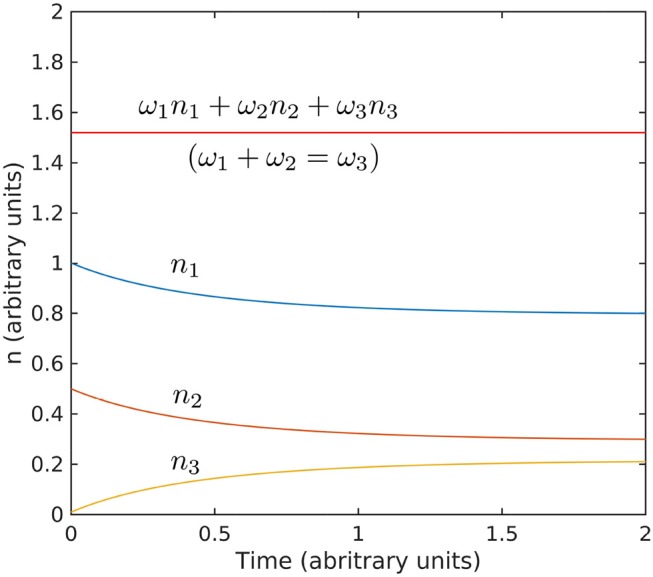
Evolution toward stationarity of the solution of the kinetic Equation 18. The initial conditions used *n*_1_(0) = 1, *n*_2_(0) = 0.5, and *n*_3_(0) = 0.01, and interaction coefficient are arbitrary and exaggerated to highlight the behavior. The Hamiltonian ω_1_*n*_1_ + ω_2_*n*_2_ + ω_3_*n*_3_ is conserved for ω_1_ + ω_2_ = ω_2_ (values for ω are also arbitrary).

Applied to a theta-gamma triad (γ, θ, γ + θ), κ_1_ = *k*_θ_, κ_2_ = *k*_γ_, and κ_3_ = *k*_θ_ + *k*_γ_ (yellow dot in Figure 8), this result is consistent with the biphase value of π in Figure 5B, upper-right panel. It is easy to check that effect of this type of coupling is that gamma envelope is maximal in theta troughs. Indeed, let φ_*j*_ = *a*_*j*_ cos [κ_*j*_*x* − ω_*j*_*t* + ψ_*j*_], where κ_*j*_ and ω_*j*_ satisfy the resonance conditions 16. Elementary trigonometric manipulations yield the gamma envelope $\propto \sin\left(\frac{\omega_{1}}{2}t\right).$ In other words, the gamma envelope is in quadrature with theta and its period is twice that of theta. The kinetic evolution of a triad is illustrated in Figure 9. The interactions described by the three-wave model result in time-reversible cyclic transfers of energy (the total energy is conserved if the system is at resonance). In contrast, the long-time, *average* behavior (Equation 18) shows a slow irreversible trend toward the stationary solution. If a full set of triads is taken into account, because all triads interact, a weak energy flow (turbulent cascade) develops that has the effect of driving the system of modes toward stationary distribution of energy over scales (RJ spectrum). If a sink is introduced in the small scales (e.g., high frequencies), the flow will naturally be directed toward the leaking mode, where it is taken out of the system. In this case a stationary state can be maintained only if energy is pumped into the system at the leakage rate, and a stationary state is realized if the energy is injected by the source at the rate it is lost to the sink. The cross-scale flow of energy is in this case constant at all scales. This type of stationarity corresponds to the KZ spectra.

## 4. Discussion

While a wealth of knowledge has accumulated in recent years about brain activity at microscopic and macroscopic scales, the need for a consistent theory of the dynamics of intermediate-scale (mesoscopic) processes has received comparatively little attention, possibly because, in some parts of the nervous system, they are the expression of activity of complex microscopic structures (e.g., for example central pattern generators can maintain specific, yet re-configurable rhythms; e.g., Rabinovich et al., 2012; Gutierrez et al., 2013; Marder et al., 2016). Although some microcircuits, with definite structure, may be able to impose an oscillation on the mesoscale, the cortex, with an isotropic and homogeneous structure, is rhythmically organized in a different manner, with a different source and might play a different role.

A number of previous studies (Lashley, 1942; Hebb, 1958; Freeman, 2000a, 2007; Freeman and Vitiello, 2010) hypothesize that mesoscopic processes in the cortex represent the essential cognition step of abstraction and generalization, and therefore provide an essential mechanism for integration of brain activity at all scales. This observation is particularly intriguing, because the isotropic and homogeneous structure of the cortex at the anatomical mesoscale (e.g., any highly recurrent region in which activity is projected back into the same region or dense inter-connectivity mediated by interneurons; Lorente de No, 1938; Freund and Buzsáki G., 1996; Buzsáki et al., 2004; Mante et al., 2013) suggests that the material support of activity in the temporal mesoscale (e.g., gamma frequency band) is collective neural activity, Freemans's (2000a) “mass action”. These ideas suggest that the physics of mesoscopic collective action in the cortex is intimately related to cognition; that *the physics of collective action is, in fact, the physics of cognition*.

The focus of this study is the dynamics of mesoscopic collective action and its role in the general energy balance in the brain. Despite the potentially paramount importance of the topic, studies dedicated to its physics are few (but of outstanding quality; e.g., Wilson and Cowan, 1972, 1973; Wright and Liley, 1995; Troy, 2008; Cowan et al., 2016). Here, we attempt to lay the foundation of a systematic theory of collective action. A few recent studies show that collective action in the hippocampus takes the form of propagating waves (Lubenov and Siapas, 2009; Patel et al., 2012, 2013; Muller et al., 2018; Zhang et al., 2018), but in general, information about its spatio-temporal organization is scarce.

The discovery of the strong correlation between rat speed during active exploration and hippocampal activity provides a parametrization of the evolution of hippocampal activity with behavior. Our observations of scale distribution of LFP power (spectra) and the leading order estimators of cross-phase coupling of LFP oscillations (bispectra) in the lacunosum moleculare layer show a strong and remarkably ordered evolution (representative of both CA1 and dentate gyrus layers). The LFP power and phase coupling in the theta and gamma frequency bands increases consistently with speed. The lowest levels of LFP power are associated with a featureless power law distribution. The high-power spectra exhibit distinctive spectral peaks at theta frequency and harmonics, and significantly increased levels of gamma activity. In the transition, at intermediate states, the spectrum tilts to a smaller-slope shape that extends progressively into the gamma range generating the appearance of a spectral front. The existence of a non-zero energy, lowest-level spectrum in the absence of coherent collective action is strongly suggestive of a self-organized critical background state (Buzsaki, 2006). As collective action energy increases with exploration, the decreasing spectral slope, the appearance of a spectral front, and the development of a broad gamma peak, are strongly suggestive of a transfer of energy from the low frequencies (large scales) toward high frequencies (microscopic scales; Buzsáki and Draguhn, 2004). The similarity of collective action with the general turbulence theory is striking. This motivated us to propose weak turbulence as an framework for collective action dynamics.

In summary, we propose that the mesoscopic hippocampus may be described as an isotropic and homogeneous, active network containing a macroscopic number of randomly and densely connected neural units (neurons). Collective action represents a perturbation of a background state of the active network, that may be represented as a self-organized critical state. Because collective action is macroscopic with respect to neural units, we postulate therefore that it is fundamentally stochastic (the precise state of a single unit does not matter), weakly non-linear, and weakly dissipative. These form a minimal set of features for the development of a turbulence theory of collective action. We summarize the principles of the turbulence framework and demonstrate its applicability to observations by showing that the reduced (universal) three-wave interaction toy model provides results consistent with observations.

Our proposed description provides a unified view of the physics of active networks that reconciles the theories of self organized criticality and turbulence in the hippocampus and perhaps other regions of the cortex. The central idea of the turbulence theory is the energy cascade through the inertial range of scales. As a theory of the internal balance of a multi-scale system, a full turbulence formulation of hippocampal collective action should provide answers to questions raised by the evolution the scale distribution of LFP power. Collective action physics appears to be consistent with the energy cascade concept (Richardson, 1922; Kolmogorov, 1941; Zakharov et al., 1992; Newell et al., 2001; Nazarenko, 2011). In particular, the spectral tilting (slope change) observed in the evolution from the background state to high-speed states suggests a transition from self-organized criticality to some type of stationary turbulent state of the Kolmogorov-Zakharov kind (Zakharov and Filonenko, 1967a,b; Zakharov et al., 1992; Newell et al., 2001). Do spectral slopes change because the system transitions from a SOC background state to a turbulent, stationary state supporting non-zero cross-scale energy fluxes? Are non-linear resonances band-limited, for example, forcing theta to develop self interaction, while allowing theta-gamma resonances? Is the growth of gamma power related to a spectral bottleneck (a break in cross-scale non-linear energy exchange at high frequencies)? Further research is needed to understand the structure of this evolution; a systematic exploration of hippocampal dynamics starting from first-principles governing equations is ongoing. The scope of this study is limited to investigating the effect of running speed as a proxy for energy into the system. Future experimental applications should explore transitions between sleep and wakefulness, the effects of learning and memory recall as well as the system is compromised in aging and disease. As cognition is the consequence of activity moving through the circuits of the brain, any experimental approach which alters the forcing or how the local network capture the activity (Bottleneck, Figure 7) will have the ability to test the predictions of the turbulence theory.

Remarkably, the isotropic and homogeneous mesoscale structure appears to be a general theme that is used across species and brain regions (Lorente de No, 1938; Parent and Hazrati, 1995; Marder and Bucher, 2001; Garamszegi and Eens, 2004; Apps and Garwicz, 2005; Mante et al., 2013), which suggests a “universal computational principle” with a comprehensive reconfiguration potential, especially under *a priori unknown* conditions (Sussillo and Abbott, 2009). The nature of this computation process is not well-understood. As often argued (e.g., Freeman, 2000b; Edelman and Gally, 2001; Frisch, 2014), cognition processes cannot resemble a human-engineered system, built based on principles such as maximum simplicity, well-defined internal interactions, explicit assignment of function, no irrelevancy, and no adventitious compensation for error. Rather, they are expected to resemble biological systems: no design, no *a priori* function, and for which irrelevance has no meaning (Edelman and Gally, 2001). As Frisch (2014) states it, “biological systems have an intrinsic ability to maintain functions in the course of structural changes”, such that “specific functions can obviously be constituted on the basis of structurally different elements, a biological property that is referred to under the term degeneracy (Edelman and Gally, 2001)”.

This begs the question, if collective action is fundamental for cognition, what is its role? We conclude this study by suggesting a possible answer. An intriguing paradigm of the computational function of mesoscale turbulence is offered by Liquid State Machines (LSM) models (Jager, 2002; Maass et al., 2002). LSMs are online neural network models that process a time-windowed signal in real time. Their basic function is to perform a non-linear transformation of the input, e.g., expand it into a wave field, and hold this information for a short duration, while output neurons extract local information from the field. Learning is achieved at the readout stage. Fading memory and input separability imply that LSMs are universal function approximators, and can serve as effective online classification pre-processor for the readout neurons. If the brain is a prediction machine, scrambling to assign meaning to streams of data^10^ in real time, processing of fragmentary information (short-time windows) is crucial. A LSM may perform fast, online, short-term memory pre-processing; learning is performed by long-term memory readouts, that can record optimal responses. It is conceivable that the hippocampus uses collective action in a way similar to a LSM, perhaps as a fast online classification machine, or as a dynamical system simulator. It seems plausible to imagine the cortex as a network of LSMs. In the least, the concept seems to agree with most natural systems.

## Author Contributions

AS and AM collaborated closely in developing the turbulence framework for brain activity. AS developed part of the numerical methods and the Matlab® tools used fort the data analysis. YZ and YQ developed part of the numerical methods, and performed the numerical analysis. JK managed data collection. AM provided the impetus for the research; designed, organized, and supervised the data collection, and provided the guidance and advice regarding all things neuroscience. AS wrote the manuscript with input from all authors.

### Conflict of Interest Statement

The authors declare that the research was conducted in the absence of any commercial or financial relationships that could be construed as a potential conflict of interest.

## Footnotes

^1^Frequency bounds given here are just convention, and could be modified depending on the processes examined.

^2^While action potentials are the fundamental process that carry the signal, like any natural process, it burns more energy than it uses. Stated differently, it is not a lossless system. Rather, synaptic transmission and action potentials comes at the price of ion exchange/energy loss (ATP required to maintain membrane charge).

^3^The homogeneous and isotropic character of the hippocampal CA1 is illustrated by the randomness and density of connections: ~ 390,000 neurons (Witter and Amaral, 2004), with approximately 30,000 putative excitatory synapses and ~ 1,700 inhibitory synapses onto a single pyramidal cell (Megías et al., 2001), also connected to interneurons (Freund and Buzsáki G., 1996; Marshall et al., 2002) which in turn contact several hundred principal neurons (Sik et al., 1995). When mapping out the local, circuits of the hippocampus, what is found is that the axonal connections would not necessary travel from one brain region to the next. Rather, it exhibits connectivity by which activity may locally reverberate. Lorente de No (1938); Hebb (1949); Maurer (2018).

^4^The phrase “collective activity” is equivalent to Freeman's (1975) “mass action” concept. We prefer “collective” over “mass”, because the word mass has a reserved meaning in physics.

^5^Freeman and Vitiello (2010): The problem was clearly stated over 50 years ago: “Generalization is one of the primitive basic functions of organized nervous tissue. Here is the dilemma. Nerve impulses are transmitted ⋯ from cell to cell through definite intercellular connections. Yet all behavior seems to be determined by masses of excitation. ⋯ What sort of nervous organization might be capable of responding to a pattern of excitation without limited specialized paths of conduction? The problem is almost universal in the activities of the nervous system (Lashley, 1942)”.

^6^The reader might find the history of the debate surrounding the work of Boltzmann (1872, 2003) and the birth statistical mechanics quite instructive (e.g., Pathria and Beale, 2011).

^7^The turbulence theory describes the evolution of other conserved quantities also. In fact most systems have multiple conservation laws (e.g., Nazarenko, 2011).

^8^Other scenarios are easy to imagine and have been observed. For example, if the dissipation capacity off the sink is smaller than the input rate at the source, energy will accumulate in the small scale range, possibly causing some system failure (e.g., a switch to different physical regime). This is a type of bottleneck scenario (e.g., L'vov et al., 2007; Meyers and Meneveau, 2008; Proment et al., 2009; Nazarenko, 2011).

^9^Scale localization of dissipation processes is common in physics. In the case of water waves, for example, long swells (ocean waves people surf; wavelength ~ 100 m) lack a direct dissipation mechanism and can propagate for hundred of kilometers with negligible decay, while capillary waves (wavelength ~ cm) experience strong dissipation. Because energy cannot jump scales from swells directly to capillary waves, swells decay by cascading their energy into progressively smaller scales until the dissipation scale is reached.

^10^In the “guess the phrase” game, the letters of a common phrase, initially blank, are filled out randomly; the goal is to identify the full sequence the fastest.

## References

[B1] Ablowitz M. Segur H. (1981). Soltons and the Inverse Scattering Transform. Philadelphia, PA: SIAM.

[B2] Ahmed O. J. Mehta M. (2012). Running speed alters the frequency of hippocampal gamma oscillations. J. Neurosci. 32, 7373–7383. 10.1523/JNEUROSCI.5110-11.2012 22623683 PMC3366345

[B3] Alexeev B. V. (2004). Generalized Boltzmann Physical Kinetics. Elsevier.

[B4] Allen P. G. Collins F. S. (2013). Toward the final frontier: the human brain. Wall Street J.

[B5] Anenkov S. Y. Shrira V. I. (2018). Spectral evolution of weakly nonlinear random waves: kinetic description versus direct numerical simulations. J. Fluid Mech. 844, 766–795. 10.1017/jfm.2018.185

[B6] Apps R. Garwicz M. (2005). Anatomical and physiological foundations of cerebellar information processing. Nat. Rev. Neurosci. 6:297. 10.1038/nrn1646 15803161

[B7] Bak P. Tang C. Wiesenfeld K. (1988). Self-organized criticality. Phys. Rev. A 38, 364–375. 9900174 10.1103/physreva.38.364

[B8] Beggs J. M. Plenz D. (2003). Neuronal avalanches in neocortical circuits. J. Neurosci. 23, 11167–11177. 10.1523/JNEUROSCI.23-35-11167.2003 14657176 PMC6741045

[B9] Beggs J. M. Timma N. (2012). Being critical of criticality in the brain. Front. Physiol. 3:163. 10.3389/fphys.2012.00163 22701101 PMC3369250

[B10] Belluscio M. A. Mizuseki K. Schmidt R. Buzsaki G. (2012). Cross-frequency phase-phase coupling between theta and gamma oscillations in the hippocampus. J. Neurosci. 32, 423–435. 10.1523/JNEUROSCI.4122-11.2012 22238079 PMC3293373

[B11] Boltzmann L. (1872). Weitere Studien über das Wärmegleichgewicht unter Gasmolekülen. Sitzungsberichte Akademie der Wissenschaften 66, 275–370.

[B12] Boltzmann L. (2003). Further studies on the thermal equilibrium of gas molecules, in The Kinetic Theory of Gases (World Scientific), 262–349.

[B13] Boyd R. (2003). Nonlinear Optics. San Diago, CA: Academic Press.

[B14] Bragin A. Jando G. Nadasdy Z. van Landeghem M. Buzsaki G. (1995). Dentate EEG spikes and associated interneuronal population bursts in the hippocampal hilar region of the rat. J. Neurophysiol. 73, 1691–1705. 7643175 10.1152/jn.1995.73.4.1691

[B15] Buzsáki G. (1986). Hippocampal sharp waves: their origin and significance. Brain Res. 398, 242–252. 3026567 10.1016/0006-8993(86)91483-6

[B16] Buzsaki G. (2002). Theta oscillations in the hippocampus. Neuron 33, 325–340. 10.1016/S0896-6273(02)00586-X 11832222

[B17] Buzsaki G. (2006). Rhythms of the Brain. Oxford University Press.

[B18] Buzsáki G. Czopf J. Kondakor I. Kellenyi L. (1986). Laminar distribution of hippocampal rhythmic slow activity (RSA) in the behaving rat: current-source density analysis, effects of urethane and atropine. Brain Res. 365, 125–137. 3947979 10.1016/0006-8993(86)90729-8

[B19] Buzsáki G. Draguhn A. (2004). Neuronal oscillations in cortical networks. Science 304, 1926–1929. 10.1126/science.1099745 15218136

[B20] Buzsáki G. Geisler C. Henze D. Wang X. (2004). Interneuron diversity series: circuit complexity and axon wiring economy of cortical interneurons. Trends Neurosci. 27, 186–193. 10.1016/j.tins.2004.02.007 15046877

[B21] Buzsáki G. Leung W. Vanderwolf C. (1983). Cellular bases of hippocampal EEG in the behaving rat. Brain Res. 287, 139–171. 6357356 10.1016/0165-0173(83)90037-1

[B22] Chen Z. Resnik E. McFarland J. Sakmann B. Mehta M. (2011). Speed controls the amplitude and timing of the hippocampal gamma rhythm. PLoS ONE 6:e21408. 10.1371/journal.pone.0021408 21731735 PMC3123337

[B23] Chorbak J. J. Buzsaki G. (1998). Gamma oscillations in the entorhinal cortex of the freely behaving rat. J. Neurosci. 18, 388–398. 9412515 10.1523/JNEUROSCI.18-01-00388.1998 PMC6793397

[B24] Coppi B. Rosenbluth M. Sudan R. (1969). Nonlinear interactions of positive and negative energy modes in rarified plasmas. Ann. Phys. 55, 207–247.

[B25] Cowan J. D. Neuman J. van Drongelen W. (2016). Wilson–Cowan equations for neocortical dynamics. J. Math. Neurosci. 6:1. 10.1186/s13408-015-0034-5 26728012 PMC4733815

[B26] Craick A. (1985). Wave Interactions and Fluid Flows. Cambridge University Press.

[B27] Cross M. and Hohenberg, P. (1993). Pattern formation outside of equilibrium. Rev. Modern Phys. 65, 851–1112.

[B28] Edelman G. Gally J. (2001). Degeneracy and complexity in biological systems. Proc. Natl. Acad. Sci. U.S.A. 98, 13763–13768. 10.1073/pnas.231499798 11698650 PMC61115

[B29] Elgar S. (1987). Relationships involving third moments and bispectra of a harmonic process. IEEE Trans. Acoust. Speech Signal Process. 1725–1726.

[B30] Elgar S. Guza R. T. (1985). Observations of bispectra of shoaling surface infragravity waves. J. Fluid Mech. 161, 425–448.

[B31] Ermentrout G. (1981). Stable small-amplitude solutions in reaction-diffusion systems. Q. Appl. Math. 39, 61–86.

[B32] Ermentrout G. Chen X. Chen Z. (1997). Transition fronts and localized structures in bistable reaction-diffusion equations. Phys, D 108, 147–167.

[B33] Fechner G. T. (1860). Elemente der Psychophysik. Breitkopf und Härtel.

[B34] Fernández-Ruiz A. Oliva A. Nagy G. A. Maurer A. P. Berenyi A. Buzsaki G. (2017). Entorhinal-CA3 dual-input control of spike timing in the hippocampus by theta-gamma coupling. Neuron 93, 1213–1226. 10.1016/j.ijpsycho.2005.12.009 28279355 PMC5373668

[B35] Freeman W. J. (1975). Mass Action in the Nervous System. New York, NY: Academic Press.

[B36] Freeman W. J. (2000a). Neurodynamics: An Exploration in Mesoscopic Brain Dynamics. London: Springer-Verlag.

[B37] Freeman W. J. (2000b). A proposed name for aperiodic brain activity: stochastic chaos. Neural Netw. 13, 11–13. 10.1016/S0893-6080(99)00093-3 10935453

[B38] Freeman W. J. (2006). A cinematographic hypothesis of cortical dynamics in perception. Int. J. Psychophysiol. 60, 149–161. 10.1016/j.ijpsycho.2005.12.009 16513196

[B39] Freeman W. J. (2007). Definitions of state variables and state space for brain-computer interface : Part 1. Multiple hierarchical levels of brain function. Cogn. Neurodyn. 1, 3–14. 10.1007/s11571-006-9001-x 19003492 PMC2288954

[B40] Freeman W. J. Vitiello G. (2010). Vortices in brain waves. Int. J. Modern Phys. B 24, 3269–3295. 10.1142/S0217979210056025

[B41] Freund T. Buzsáki G (1996). Interneurons of the hippocampus. Hippocampus 6, 347–470. 8915675 10.1002/(SICI)1098-1063(1996)6:4<347::AID-HIPO1>3.0.CO;2-I

[B42] Frisch S. (2014). How cognitive neuroscience could be more biological—and what it might learn from clinical neuropsychology. Front. Hum. Neurosci. 8:541. 10.3389/fnhum.2014.00541 25100981 PMC4104996

[B43] Garamszegi L. Eens M. (2004). The evolution of hippocampus volume and brain size in relation to food hoarding in birds. Ecol. Lett. 7, 1216–1224. 10.1111/j.1461-0248.2004.00685.x

[B44] Gibbs J. W. (1902). Elementary principles in Statistical Mechanics. Longmans, Green and Co.

[B45] Green J. D. Arduini A. (1954). Hippocampal electrical activity in arousal. J. Neurophysiol. 17, 533–557. 13212425 10.1152/jn.1954.17.6.533

[B46] Green J. D. Machne X. (1955). Unit activity of rabbit hippocampus. Am. J. Physiol. 181, 219–224. 14376599 10.1152/ajplegacy.1955.181.2.219

[B47] Gutierrez G. J. O'Leary T. Marder E. (2013). Multiple mechanisms switch an electrically coupled, synaptically inhibited neuron between competing rhythmic oscillators. Neuron 77, 845–858. 10.1016/j.neuron.2013.01.016 23473315 PMC3664401

[B48] Hasselmann K. Munk W. McDonald G. (1963). Bispectra of ocean waves, in Proceedings of Symposium on Time Series Analysis, ed Rosenblatt M. (New York, NY: John Wiley), 125–139.

[B49] Haubrich R. A. MacKenzie G. S. (1965). Earth noise, 5 to 500 millicycles per second: 2. Reaction of the Earth to oceans and atmosphere. J. Geophys. Res. 70, 1429–1440.

[B50] Hebb D. (1949). The Organization of Behavior: A Neuropsychological Theory. New York, NY: Wiley.

[B51] Hebb D. (1958). A Textbook of Psychology. Philadelphia, PA: W. B. Saunders.

[B52] Hirase H. Czurko A. Csicsvari J. Buzsaki G. (1999). Firing rate and theta-phase coding by hippocampal pyramidal neurons during ‘space clamping’. Eur. J. Neurosci. 11, 4373–4380. 10594664 10.1046/j.1460-9568.1999.00853.x

[B53] Hyafil A. (2015). Misidentifications of specific forms of cross-frequency coupling: three warnings. Front. Neurosci. 9:370. 10.3389/fnins.2015.00370 26500488 PMC4598949

[B54] Jager H. (2002). Short Term Memory in Echo State Networks. Technical Report GMD Report 152, German National Research Center for Information Technology.

[B55] Kemere C. Carr M. Karlsson M. Frank L. (2013). Rapid and continuous modulation of hippocampal network state during exploration of new places. PLoS ONE 8:e73114. 10.1371/journal.pone.0073114 24023818 PMC3759452

[B56] Khinchin A. I. (1949). Mathematical Foundations of Statistical Mechanics. Dover Publications Inc.

[B57] Kolmogorov A. N. (1941). The local structure of turbulence in incompressible viscous fluid for very large Reynolds numbers, in Proceedings: Mathematical and Physical Sciences: Turbulence and Stochastic Process: Kolmogorov's Ideas 50 Years On, 9–13.

[B58] Kovach C. K. Oya H. Kawasaki H. (2018). The bispectrum and its relationship to phase-amplitude coupling. Neuroimage 173, 518–539. 10.1016/j.neuroimage.2018.02.033 29481965

[B59] Kropff E. Carmichael J. Moser M. Moser E. (2015). Speed cells in the medial entorhinal cortex. Nature 523, 419–424. 10.1038/nature14622 26176924

[B60] Lashley K. S. (1942). The problem of cerebral organization in vision, in Visual Mechanisms, Vol. 301, ed Klüver H. (Oxford, UK: Jacques Cattell), 301–322.

[B61] Lashley K. S. (1958). Cerebral organization and behavior. Res. Publ. Assoc. Res. Nervous Mental Dis. 6, 14–18. 13527780

[B62] Lasztóczi B. Klausberger T. (2014). Layer-specific GABAergic control of distinct gamma oscillations in the CA1 hippocampus. Neuron 81, 1126–1139. 10.1016/j.neuron.2014.01.021 24607232

[B63] Lisman J. Idiart M. (1995). Storage of 7 +/- 2 short-term memories in oscillatory subcycles. Science 267, 1512–1515. 7878473 10.1126/science.7878473

[B64] Lorente de No R. (1938). Architectonics and structure of the cerebral cortex, in Physiology of the Nervous System, ed Fulton J. L. (Oxford University Press), 291–330.

[B65] Lubenov E. V. Siapas A. (2009). Hippocampal theta oscillations are travelling waves. Nature 459, 534–539. 10.1038/nature08010 19489117

[B66] L'vov V. (1998). Universality of turbulence. Nature 396, 519–520.

[B67] L'vov V. Nazarenko S. Rudenko O. (2007). Bottleneck crossover between classical and quantum superfluid turbulence. Phys. Rev. B 76:024520 10.1103/PhysRevB.76.024520

[B68] Maass W. Natschlager T. Markram H. (2002). Real-time computing without stable states: a new framework for neural computation based on perturbations. Neural Comput. 14, 2531–2560. 12433288 10.1162/089976602760407955

[B69] Mante V. Sussillo D. Shenoy K. Newsome W. (2013). Context-dependent computation by recurrent dynamics in prefrontal cortex. Nature 503, 78–84. 10.1038/nature12742 24201281 PMC4121670

[B70] Marder E. Bucher D. (2001). Central pattern generators and the control of rhythmic movements. Curr. Biol. 11, R986–R996. 10.1016/S0960-9822(01)00581-4 11728329

[B71] Marder E. Gutierrez G. Nusbaum M. (2016). Complicating connectomes: Electrical coupling creates parallel pathways and degenerate circuit mechanisms. Dev. Neurobiol. 77, 597–609. 10.1002/dneu.22410 27314561 PMC5412840

[B72] Marshall L. Henze D. Hirase H. Leinekugel X. Dragoi G. Buzsaki G. (2002). Hippocampal pyramidal cell–interneuron spike transmission is frequency dependent and responsible for place modulation of interneuron discharge. J. Neurosci. 22, 1–5. 10.1523/JNEUROSCI.22-02-j0001.2002 11784809 PMC6758681

[B73] Masuda A. Kuo Y.-Y. (1981). A note on the imaginary part of bispectra. Deep Sea Res. 28, 213–222.

[B74] Maurer A. P. Vanrhoads S. R. Sutherland G. Lipa P. McNaughton B. (2005). Self-motion and the origin of differential spatial scaling along the septo-temporal axis of the hippocampus. Hippocampus 15, 841–852. 10.1002/hipo.20114 16145692

[B75] Maurer L. N. A. (2018). Recalling lashley and reconsolidating hebb. Hippocampus. 10.1002/hipo.23027. [Epub ahead of print]. 30216593 PMC6417981

[B76] McNaughton B. L. Barnes C. A. O'Keefe J. (1983). The contributions of position, direction, and velocity to single unit activity in the hippocampus of freely-moving rats. Exp. Brain Res. 52, 41–49. 6628596 10.1007/BF00237147

[B77] Megías M. Emri Z. Freund T. Gulyas A. (2001). Total number and distribution of inhibitory and excitatory synapses on hippocampal CA1 pyramidal cells. Neuroscience 102, 527–540. 10.1016/S0306-4522(00)00496-6 11226691

[B78] Meyers J. Meneveau C. (2008). A functional form for the energy spectrum parametrizing bottleneck and intermittency effects. Phys. Fluids 20, 065109–065107. 10.1063/1.2936312

[B79] Morris R. Hagan J. (1983). Hippocampal electrical activity and ballistic movement, in Neurobiology of the Hippocampus, ed Seifert W. (London: Academic Press), 321–331.

[B80] Muller L. Chavane F. Reynolds J. Sejnowski T. J. (2018). Cortical travelling waves: mechanisms and computational principles. Nat. Rev. Neurosci. 19, 255–268. 10.1038/nrn.2018.20 29563572 PMC5933075

[B81] Nazarenko S. (2011). Wave Turbulence. Springer-Verlag.

[B82] Newell A. Nazarenko S. Biven L. (2001). Wave turbulence and intermittency. Phys. D Nonlinear Phenomena 152-153, 520–550. 10.1016/S0167-2789(01)00192-0

[B83] Newell A. Rumpf B. (2010). Wave turbulence and intermittency. Annu. Rev. Fluid Mech. 43, 59–78. 10.1146/annurev-fluid-122109-160807

[B84] Newell A. C. (1985). Soltons in Mathemtics and Physics. Soc. for Industrial and App. Mathematics.

[B85] Nolting W. (2016). Theoretical Physics 3: Electrodynamics. Springer.

[B86] Papoulis A. Pillai S. (2002). Probability, Random Variables, and Stochastic Processes. Tata McGraw-Hill Education.

[B87] Parent A. Hazrati L.-N. (1995). Functional anatomy of the basal ganglia. i. the cortico-basal ganglia-thalamo-cortical loop. Brain Res. Rev. 20, 91–127. 7711769 10.1016/0165-0173(94)00007-c

[B88] Passot T. Newell A. (1994). Towards a universal theory for natural patterns. Phys. D 74, 301–352.

[B89] Patel J. Fujisawa S. Berenyi A. Royer S. Buzsaki G. (2012). Traveling theta waves along the entire septotemporal axis of the hippocampus. Neuron 75, 410–417. 10.1016/j.neuron.2012.07.015 22884325 PMC3427387

[B90] Patel J. Schomburg E. W. Berenyi A. Fujisawa S. Buzsaki G. (2013). Local generation and propagation of ripples along the septotemporal axis of the hippocampus. J. Neurosci. 33, 17029–17041. 10.1523/JNEUROSCI.2036-13.2013 24155307 PMC3807028

[B91] Pathria R. K. Beale P. D. (2011). Statistical Mechanics, 3rd Edn. Elsevier.

[B92] Percival D. Walden A. (2009). Spectral Analysis for Physical Applications. Cambridge: Cambridge University Press.

[B93] Petsche H. Stumpf C. (1960). Topographic and toposcopic study of origin and spread of the regular synchronized arousal pattern in the rabbit. Electroencephalogr. Clin. Neurophysiol. 12, 589–600. 14432410 10.1016/0013-4694(60)90101-2

[B94] Phillips O. (1977). The Dynamics of the Upper Ocean. Cambridge UK: Cambridge University Press.

[B95] Pollack G. Stump D. R. (2002). Electromagnetism. Addison Wesley.

[B96] Priestley J. (1981). Spectral Analysis and Time Series. Academic Press.

[B97] Proment D. Nazarenko S. Onorato M. (2009). Quantum turbulence cascades in the Gross-Pitaevskii model. Phys. Rev. A 80:051603 10.1103/PhysRevA.80.051603

[B98] Pruessner G. (2012). Self-Organized Criticality, Theory, Models, and Characterization. Cambridge University Press.

[B99] Rabinovich M. Trubetskov C. (1989). Oscillations and Waves in Linear and Nonlinear Systems. Kluwer Academic Publishers.

[B100] Rabinovich M. I. Friston K. J. Varona P. (2012). Principles of Brain Dynamics. The MIT Press.

[B101] Richardson L. (1922). Weather Prediction by Numerical Process. Cambridge University Press.

[B102] Rivas J. Gatzelu J. Garcia-Austt E. (1996). Changes in hippocampal cell discharge patterns and theta rhythm spectral properties as a function of walking velocity in the guinea pig. Exp. Brain Res. 108, 113–118. 8721159 10.1007/BF00242908

[B103] Rosenblatt M. Van Ness J. (1965). Estimation of the bispectrum. Ann. Math. Stat. 36, 1120–1136.

[B104] Schomburg E. W. Fernandez-Ruiz A. Mizuseki K. Berenyi A. Anastassiou C. Koch C.. (2014). Theta phase segregation of input-specific gamma patterns in entorhinal-hippocampal networks. Neuron 84, 470–485. 10.1016/j.neuron.2014.08.051 25263753 PMC4253689

[B105] Shen J. Barnes C. McNaughton B. Skaggs W. Weaver K. (1997). The effect of aging on experience-dependent plasticity of hippocampal place cells. J. Neurosci. 17, 6769–6782. 9254688 10.1523/JNEUROSCI.17-17-06769.1997 PMC6573142

[B106] Sheremet A. Burke S. Maurer A. (2016). Movement enhances the nonlinearity of hippocampal theta. J. Neurosci. 36, 4218–4230. 10.1523/JNEUROSCI.3564-15.2016 27076421 PMC4829647

[B107] Sheremet A. Zhou Y. Kennedy J. Qin Y. Burke S. Maurer A. (2018). Theta-gamma coupling: a nonlinear dynamical model. BioRXiv 10.1101/304238

[B108] Siapas A. G. Lubenov E. Wilson M. (2005). Prefrontal phase locking to hippocampal theta oscillations. Neuron 46, 141–151. 10.1016/j.neuron.2005.02.028 15820700

[B109] Sik A. Pettonen M. Ylinen A. Buzsaki G. (1995). Hippocampal CA1 interneurons: an *in vivo* intracellular labeling study. J. Neurosci. 15, 6651–6665. 7472426 10.1523/JNEUROSCI.15-10-06651.1995 PMC6577981

[B110] Sussillo D. Abbott L. (2009). Generating coherent patterns of activity from chaotic neural networks. Neuron 63, 544–557. 10.1016/j.neuron.2009.07.018 19709635 PMC2756108

[B111] Swami A. Mendel J. Nikias C. (2001). Higher Order Spectral Analysis Toolbox: A MATLAB Toolbox for Spectral and Polyspectral Analysis, and Time-Frequency Distributions. United Signals & Systems.

[B112] Toda M. Kubo R. Saito N. (1983). Statistical Physics 1, Equilibrium Statistical Mechanics. Springer-Verlag.

[B113] Tognoli E. Kelso J. (2014). The metastable brain. Neuron 81, 35–48. 10.1016/j.neuron.2013.12.022 24411730 PMC3997258

[B114] Troy W. C. (2008). Wave phenomena in neuronal networks, in Dissipative Solitons: From Optics to Biology and Madicine, Lecture Notes in Physics 751, eds Akhmediev N. Ankiewicz A. (Springer), 431–452.

[B115] Vandecasteele M. Royer S. M. S. Belluscio M. Berrnyi A. Diba K. Fujisawa S. (2012). Large-scale recording of neurons by movable silicon probes in behaving rodents. J. Visual Exp. 61:3568 10.3791/3568 PMC3399468 22415550

[B116] Vanderwolf C. (1969). Hippocampal electrical activity and voluntary movement in the rat. Electroencephal. Clin. Neurophysiol. 26, 407–418. 4183562 10.1016/0013-4694(69)90092-3

[B117] Vertes R. P. Kocsis B. (1997). Brainstem-diencephalo-septohippocampal systems controlling the theta rhythm of the hippocampus. Neuroscience 81, 893–926. 9330355 10.1016/s0306-4522(97)00239-x

[B118] Weber H. (1860). De pulsa resorptione auditu et tactu. Annot. Anat. Physiol. 4, 176–258.

[B119] Weiland J. Wilhelmsson H. (1977). Coherent Non-linear Interaction of Waves in Plasmas. Pergamon Press.

[B120] Whishaw I. Q. Vanderwolf C. (1973). Hippocampal EEG and behavior: changes in amplitude and frequency of RSA (theta rhythm) associated with spontaneous and learned movement patterns in rats and cats. Behav. Biol. 8:461–484. 4350255 10.1016/s0091-6773(73)80041-0

[B121] Wilson H. R. Cowan J. D. (1972). Excitatory and inhibitory interactions in localized populations of model neurons. Biophys. J. 12, 1–24. 4332108 10.1016/S0006-3495(72)86068-5 PMC1484078

[B122] Wilson H. R. Cowan J. D. (1973). A mathematical theory of the functional dynamics of cortical and thalamic nervous tissue. Kybernetik 13, 55–80. 4767470 10.1007/BF00288786

[B123] Witter M. Amaral D. (2004). Chapter 21: Hippocampal formation, in The Rat Nervous System, 3rd Edn., ed Paxinos G. (Academic Press), 635–704.

[B124] Wright J. J. Liley D. T. J. (1995). Simulation of electrocortical waves. Biol. Cybernet. 72, 347–356. 7748961 10.1007/BF00202790

[B125] Ylinen A. Bragin A. Nadasdy Z. Jando G. Szabo I. Sik A. Buzsaki G. (1995). Sharp wave-associated high-frequency oscillation (200 Hz) in the intact hippocampus: network and intracellular mechanisms. J. Neurosci. 15, 30–46. 7823136 10.1523/JNEUROSCI.15-01-00030.1995 PMC6578299

[B126] Zakharov V. (1999). Statistical theory of gravity and capillary waves on the surface of a finite-depth fluid. Eur. J. Mech. B/Fluids 18, 327–344.

[B127] Zakharov V. Filonenko N. (1967a). Weak turbulence of capillary waves. J. Appl. Mech. Tech. Phys. 8, 37–42.

[B128] Zakharov V. L'vov V. Falkcovich G. (1992). Kolmogorov Spectra of Turbulence I. Springer-Verlag.

[B129] Zakharov V. E. Filonenko N. N. (1967b). Energy spectrum for stochastic oscillations of the surface of a liquid. Sov. Phys. Doklady 11, 881–884.

[B130] Zhang H. Watrous A. J. Patel A. Jacobs J. (2018). Theta and alpha oscillations are traveling waves in the human neocortex. Neuron 98, 1269–1281. 10.1016/j.neuron.2018.05.019 29887341 PMC6534129

[B131] Zheng C. Bieri K. Hwaun E. Colgin L. L. (2016). Fast gamma rhythms in the hippocampus promote encoding of novel object–place pairings. eNeuro. 3, 1–19. 10.1523/ENEURO.0001-16.2016 27257621 PMC4874540

[B132] Zheng C. Bieri K. Trettel S. Colgin L. (2015). The relationship between gamma frequency and running speed differs for slow and fast gamma rhythms in freely behaving rats. Hippocampus 25, 924–938. 10.1002/hipo.22415 25601003 PMC4499477

[B133] Zhou Y. Sheremet A. Kennedy J. Maurer A. (2018). Linear analysis of hippocampal lfp: slow gamma vs high theta. BioRXiv. 10.1101/428490

