# Supplementary material for: Wave Turbulence and Energy Cascade in the Hippocampus
Source: Front Syst Neurosci. 2019 Jan 4;12:62. doi: 10.3389/fnsys.2018.00062 (PMC6328460; doi:10.3389/fnsys.2018.00062)
Supplement: Supplementary file 1 [file Data_Sheet_1.pdf]

WAVE TURBULENCE AND ENERGY CASCADE IN THE HIPPOCAMPUS SUPPLEMENTARY MATERIAL

This is a summary of wave turbulence principles, abbreviated and simplified to retain only the main ideas. Turbulence is a rich theory, with deep implications for the physics of large systems, covering a wide range of topics that include hydrodynamics, plasma physics, nonlinear optics, aggregation-fragmentation processes, ocean waves, and many others. The goal of this succinct account is to provide a possible blueprint for future investigations in to the dynamics of mesoscale neural collective action.

1. Governing equations

Following the standard thermodynamics formalism [e.g., Callen, 1960], assume that the physical system is a one-dimensional spatial network whose mesoscopic state is completely described by a function $\phi(x,t)$ that represents the deviation from an appropriately chosen equilibrium state [e.g., Wright and Liley, 1995]. Quite generally, we will assume that φ satisfies a weakly-nonlinear, non-dissipative evolution equation that tends is linear as $\phi \to 0$, i.e.,

$$L(\partial_t, \partial_x)\phi = N(\phi^2), \tag{1}$$

$$L(\partial_t, \partial_x)\phi = 0, \text{ as } \phi \to 0. \tag{2}$$

where L and N are constant-coefficient linear and nonlinear operators in ϕ and its derivatives. Because the nonlinearity is weak, if ϕ is not too large, we can neglect nonlinear terms ϕ^m with $m > 2$. Ignoring boundary conditions, equation 1 is solved using the Fourier transform

$$\varphi(k,t) = \int_{-\infty}^{\infty} \phi(x,t)e^{-2\pi ikx}dx, \tag{3a}$$

$$\phi(x,t) = \int_{-\infty}^{\infty} \varphi(k,t)e^{2\pi ikx}dk, \tag{3b}$$

where k is the wavenumber, and the functions $e^{2\pi ikx}$ are orthogonal in the sense that

$$\int_{-\infty}^{\infty} e^{2\pi ikx}dx = \delta(k), \tag{4}$$

with δ the Dirac delta function, satisfying the sifting property $\int_{-\infty}^{\infty} f(x)\delta(x) = f(0)$. Infinitesimally close to the equilibrium state, substituting $\varphi(k,t) = A(k)e^{-2\pi ift}$ into equation 2 yields the dispersion relation between the frequency f and wavenumber k [Whitham, 1974]

$$L\,(ik, -if) = 0, \tag{5}$$

which may be solved to obtain $f = f(k)$. The roots of the dispersion relation 5 are called modes. Neglecting dissipation implies that both f and k are real (with at most negligible imaginary parts). The only assumption we make about the dispersion relation is that the function $f(k)$ (or $k(f)$) is monotonically increasing for positive wavenumbers and frequencies (higher frequencies correspond to higher wavenumbers and smaller scales, i.e., shorter waves).

2. Dynamical equation in the Fourier (scale) space

A plausible way to introduce a Hamiltonian description is as follows. Assume that the function ϕ may be expressed as as function of r extensive state variables, $\phi = \phi(q)$, where $q(x,t) = (q_1, q_2, \cdots, q_r)(x,t)$. The function ϕ might be related to the local electrical field potential, and state variables might be physical space densities that describe mesoscale activity, such as number of neuronal firing pulses per unit network length, number of excitatory pulses received per unit network length, and so on. If ϕ completely characterizes the thermodynamics of the system, it determines all relevant intensive variables $p(x,t) = (p_1, p_2, \cdots, p_r)(x,t)$, through the standard thermodynamic relations

$$p = \frac{\partial \phi}{\partial q} = \left(\frac{\partial}{\partial q_1}, \frac{\partial}{\partial q_2} \cdots, \frac{\partial}{\partial q_r} \right) \phi. \tag{6}$$

Let

$$H(p,q) = 0, \tag{7}$$

be an equation of state, where H is a function of the extensive/intensive thermodynamic parameters q and p. Equation 7 is a partial differential equation for ϕ (by substitution of p from equation 6). One can readily verify by substitution that the solution to equation 7 is given by the equations

$$\frac{dq}{dt} = \frac{\partial H;}{\partial p} \frac{dp}{dt} = -\frac{\partial H}{\partial q}, \tag{8}$$

where t parameterizes the evolution of the system. Equations 8 may be interpreted as Hamilton's canonical equations, describing the evolution of the system in the space defined by the generalized coordinates q and momenta p, and subject to the constraint $H = 0$, where $H$is recognized as the Hamiltonian of the system [e.g., Peterson, 1979, Rajeev, 2008, Baldiotti et al., 2016]. A change of variables (p, q) that preserves the form of the canonical equations 8 is called a canonical transformation. The symmetry of the Hamiltonian description may be used to further simplify the dynamical equations by performing two canonical transformations: the so-called Bogoliubov transformation [e.g., Zakharov et al., 1992] $(q(x,t), p(x,t)) \rightarrow (A(x,t), A^*(x,t))$

$$A = \frac{q}{\sqrt{2}\Lambda} + i\frac{\Lambda p}{\sqrt{2}}; \; A^* = \frac{q}{\sqrt{2}\Lambda} + i\frac{\Lambda p}{\sqrt{2}}, \tag{9}$$

where the constant Λ is used to bring the variables q and p to the same physical units, followed by a Fourier transformation (the transform 3a-3b is canonical and unitary) $A(x,t) \rightarrow a(k,t)$, where

$$a(k,t) = \int_{-\infty}^{\infty} A(x,t) e^{-2\pi i k x} dx. \tag{10}$$

In the new variables, the general form of the Hamiltonian corresponding to equation 1, without creation-anihilation terms, [e.g., Zakharov et al., 1992], and retaining only the leading order nonlinearity, is

$$H = \int_{-\infty}^{\infty} \omega_k a_k a_k^* dk + \frac{1}{2} \iiint_{-\infty}^{\infty} \left(V_{1;23} a_1^* a_2 a_3 + V_{1;23}^* a_1 a_2^* a_3^* \right) \delta_{1;23}^k dk_{123}. \tag{11}$$

To simplify the notation we used the following shorthand notations: $a_j = a\left(k_j, t\right)$, $\omega_j = 2\pi f(k_j)$; and $a_k = a(k,t)$, $\omega_k = \omega(k,t)$ for modes k and k_j; $dk_{23} = dk_2 dk_3$, $\delta_{1;23}^k = \delta\left(k_1 - k_2 - k_3\right)$.

The nonlinear term is a convolution integral of all the nonlinear terms. The interaction coefficients $V_{1;23} = V(k_1, k_2, k_3)$ depend on the wavenumber, and are symmetric in the indices 2 and 3. If the Hamiltonian is identified with the energy of the system (conserved), the quantity $|a|^2$ has dimensions of action (energy × time).

The Hamiltonian form 11 is universal; the details of the physics of the system are contained in the dispersion relation $\omega = \omega(k)$ and the structure of the interaction coefficient $V_{1;23}$. The canonical equations become

$$i\overset{\bullet}{a}_k = \frac{\delta H}{\delta a_k^*}; \quad -i\overset{\bullet}{a}_k^* = \frac{\delta H}{\delta a_k}, \tag{12}$$

where we used the bullet notation for time derivative $\overset{\bullet}{q} = \frac{dq}{dt}$, and introduced the standard notation δ for the variational derivative. Because the two equations are obtained from each other through complex conjugation, the system 8 is now reduced to equation a single equation (the second equation is simply its complex conjugate). Substituting the Hamiltonian 11 into equations 12 obtains equation

$$i\overset{\bullet}{a}_k = \omega_k a_k + \frac{1}{2}\iint_{-\infty}^{\infty} \left(V_{k;12} a_1 a_2 \delta^k_{k;12} + 2V^*_{1;k2} a_1 a_2^* \delta^k_{1;k2} \right) dk_{12} + \ldots \tag{13}$$

(equation 3.1 in the paper). Equation 13, usually referred to as the dynamical equation, is the basis of our framework, and the main object of this discussion. Under the assumptions made so far, like the Hamiltonian form 11, equation 13 is universal, with the physics of the system contained in the coefficients.

3. Averaging: the BBGKY hierarchy and the kinetic equation

The goal of averaging of dynamical equation 13 is to derive evolution equations for moments of the probability distribution of φ, or alternatively, its cumulants. In the Fourier space, this is equivalent to deriving the evolution equations for quantities known as "correlators", such as $\langle a_1^* a_2 \rangle$, $\langle a_1^* a_2 a_3 \rangle$, $\langle a_1^* a_2^* a_3 a_4 \rangle$, and so on, where the angular brackets denote the ensemble average. While the derivation is of the equation is straightforward, the resulting system is comprised of an infinite sequence of equations that, at each order, involve correlators of higher order, e.g.,

$$\langle a_1^* a_2 \rangle^{\bullet} = F_2\left(\langle a_1^* a_2 a_3 \rangle\right), \; \langle a_1^* a_2 a_3 \rangle^{\bullet} = F_3\left(\langle a_1^* a_2^* a_3 a_4 \rangle\right), \; \ldots \tag{14}$$

and so on, where F_2 and F_3 are some functions. System 14, known as the BBGKY hierarchy (Bogolyubov-Born-Green-Kirkwood-Yvon; e.g., Montgomery and Tidman, 1964, Alexeev, 2004), is not closed and cannot be solved, unless some means of truncating it (closure) are found. The closure problem is familiar to statistical mechanics. We provide here a sketch of the calculations, following procedures detailed in Newell, 1999, Newell et al., 2001, Zakharov et al., 1992, Zakharov, 1999, Nazarenko, 2011 and others.

Assuming spatial homogeneity implies that

$$\langle a_1^* a_2 \rangle = n(k_1)\delta(k_1 - k_2) = n_1 \delta^k_{1;2}, \tag{15}$$

$$\left\langle a_k^* a_1 a_2 \right\rangle = \mathscr{B}_{k;12}\delta(k - k_1 - k_2) = \mathscr{B}_{k;12}\delta^k_{k;12}. \tag{16}$$

(equations 3.4-3.5 in the paper), where quantity $n(k)$ represents the action density, but is also referred to as "occupancy number" or "number of particles", by analogy with quantum

mechanics. We will also call n and $\mathscr{B}$ by their generic stochastic-process names of "spectrum" and "bispectrum", respectively.

Double correlator (spectrum). To derive an equation for the double correlator multiply equation by A_1^* and subtract from it its complex conjugate and average using the spatial homogeneity assumptions (equations 15- 16) obtains the lowest order equation of the BBGKY hierarchy,

$$\dot{n}_k = \iint_{-\infty}^{\infty} \left(\Im\left\{ V_{k;12} \mathscr{B}_{k;12} \delta^k_{1;k2} \right\} + 2\Im\left\{ V^*_{1;k2} \mathscr{B}^*_{1;k2} \delta^k_{1;k2} \right\} \right) dk_{12} \tag{17}$$

describing the evolution of the spectrum n as a function of the bispectrum.

Triple correlator (bispectrum). An equation for the evolution of the bispectrum $\mathscr{B}$ can also be derived from the dynamical equation 13. Differentiating to time the triple products $a_k^* a_1 a_2$ and $a_1 a_k^* a_2^*$ and averaging yields, for example for the first product in the equation for the double correlator

$$\begin{aligned} \left(a_1 a_k^* a_2^*\right)^{\bullet} \delta^k_{1;k2} &= i\Delta^{\omega}_{k;12} \left\langle a_1 a_k^* a_2^* \right\rangle \delta^k_{1;k2} \\ -\frac{i}{2} \iint_{-\infty}^{\infty} &\left(V_{1;34} \left(a_k^* a_2^* a_3 a_4\right) \delta^k_{1;34} + 2V^*_{3;14} \left(a_k^* a_2^* a_3 a_4^*\right) \delta^k_{3;14} \right) dk_{34} \\ +\frac{i}{2} \iint_{-\infty}^{\infty} &\left(V^*_{k;34} \left(a_1 a_2^* a_3^* a_4^*\right) \delta^k_{k;34} + 2V_{3;k4} \left(a_1 a_2^* a_3^* a_4\right) \delta^k_{3;k4} \right) dk_{34} \\ +\frac{i}{2} \iint_{-\infty}^{\infty} &\left(V^*_{2;34} \left(a_1 a_k^* a_3^* a_4^*\right) \delta^k_{2;34} + 2V_{3;24} \left(a_1 a_k^* a_3^* a_4\right) \delta^k_{3;24} \right) dk_{34}, \end{aligned} \tag{18}$$

showing that the evolution of triple correlators is driven by quadruple correlators $\langle a_k a_1 a_2^* a_3^* \rangle$. The same procedure can be applied to derive evolution equations for higher-order correlators. At each step next order correlators are involved, thus the resulting system of equations is not "closed" and cannot be solved. Assumptions that lead to the closure of the system have to be made.

3.1. **Quasi-Gaussian closure.** If amplitudes are small and stay small through the evolution process, say $a = O(\epsilon)$, where $\epsilon \ll 1$, then correlators are (and stay) well ordered, i.e., $\langle a_1 a_2 \rangle = O(\epsilon^2)$, $\langle a_1^* a_2 a_3 \rangle = O(\epsilon^3)$,and so on. This property of the equation is commonly referred to as weak nonlinearity. Then fourth-order correlators $\langle a_1 a_2 a_3^* a_4^* \rangle$ should have a generic quasi-Gaussian structure (i.e., dominated by variance, e.g., Newell et al. 2001, Nazarenko 2011),

$$\langle a_1 a_2 a_3^* a_4^* \rangle = n_1 n_2 \delta_{1;3} \delta_{2;4} + n_1 n_2 \delta_{1;3} \delta_{2;4} + Q_{1234} \tag{19}$$

where Q_{1234} is a irreducible residual of higher order (ϵ^{-5}). Substituting equation 19 into the average of the triple product in equation 18 and neglecting the irreducible terms yields the equation for the evolution of the bispectrum

$$\left(i\frac{d}{dt} + \Delta^{\omega}_{k;12} \right) \mathscr{B}_{k;12} = -V^*_{k;12} \delta^k_{k;12} n_k n_1 n_2 \left(\frac{1}{n_k} - \frac{1}{n_2} - \frac{1}{n_2} \right). \tag{20}$$

Equations 17 and 20 form the system of coupled equations (equations 3.6 in the paper). The BBGKY system is closed, since the evolution of the bispectrum depends only on the spectrum. Equations 17 and 20 describe the stochastic evolution of the system, on time scales of order $O\left(\epsilon^{-4}\right)$.

We should stress that this closure is valid only for as long as the correlators are well ordered. If singularities appear as a result of the evolution, the closure breaks down. Simple scaling consideration [Newell and Rumpf, 2010] show that the closure is scale dependent and necessarily breaks a small scales.

3.2. **The kinetic equation.** With some standard simplifications [e.g., Zakharov et al., 1992, Zakharov, 1999, Anenkov and Shrira, 2018], the system 17-20 may be simplified further. Assuming that the spectrum varies with time much slower than the linear phase ($\dot{n} \ll \Delta^{\omega}_{1;23}$; averaging the modulus squared $n = \left\langle |a|^2 \right\rangle$ eliminates the fast oscillatory time-dependence), equation 20 can be integrated approximately for $t = O\left(\frac{1}{\Delta^{\omega}_{3;12}}\right)$ to obtain

$$\mathscr{B}_{k;12} = \mathscr{B}_{k;12}(0) + iV^*_{k;12}\delta^k_{k;12}\int_0^t ds J_{k;12} e^{-i\Delta^{\omega}_{k;12}(s-t)}; \tag{21}$$

$$J_{k;12} = n_k n_1 n_2 \left(\frac{1}{n_k} - \frac{1}{n_2} - \frac{1}{n_2}\right). \tag{22}$$

If the initial bispectrum is zero ($\mathscr{B}_{k;12}(0) = 0$) and factoring out of the integral the expression $J_{k;12}$ containing the spectra obtains

$$\mathscr{B}_{k;12} = -V^*_{k;12}\delta^k_{k;12}J_{k;12}\left(\frac{1-\cos\Delta^{\omega}_{k;12}t}{\Delta^{\omega}_{k;12}} - it\,\mathrm{sinc}\,\Delta^{\omega}_{k;12}t\right), \tag{23}$$

where $\mathrm{sinc}\,x = \frac{\sin x}{x}$. The long time limit $t \to \infty$ of equation ,

$$\lim_{t\to\infty}\mathscr{B}_{k;12} = -V^*_{k;12}\delta^k_{k;12}J_{k;12}\left[\frac{P}{\Delta^{\omega}_{k;12}} - i\pi\delta\left(\Delta^{\omega}_{k;12}\right)\right] \tag{24}$$

where $\frac{P}{x}$ is Sokhozki's generalized function [Vladimirov, 2002], satisfying the relation

$$\int_{-\infty}^{\infty}\left(\frac{P}{x}\right)f(x)dx = P\int_{-\infty}^{\infty}\frac{f(x)}{x}dx, \tag{25}$$

with P denoting the principal value of the integral. This solution is meaningful only if $\Delta^{\omega}_{3;12} = 0$, a condition that comes in addition to the selection criterion $\Delta^{k}_{3;12} = 0$ for the triad. The system of equations

$$k - k_1 - k_2 = 0, \tag{26a}$$

$$\omega(k) - \omega(k_1) - \omega(k_2) = 0, \tag{26b}$$

is called "resonance conditions".

Substituting into the equation for the evolution 17 of the spectrum, which requires only the imaginary part of the bispectrum obtains, after symmetrization, a single equation called the *kinetic* equation (equation 3.7 in the paper)

$$n_k = \pi\left(\mathscr{R}_{k;12} - \mathscr{R}_{1;k2} - \mathscr{R}_{2;k1}\right), \tag{27}$$

$$\mathscr{R}_{k;12} = \iint_{-\infty}^{\infty}\left|V_{k;12}\right|^2 n_k n_1 n_2\left(\frac{1}{n_k} - \frac{1}{n_2} - \frac{1}{n_2}\right)\delta^k_{k;12}\delta^{\omega}_{k;12}dk_{12}, \tag{28}$$

where $\delta^{\omega}_{k;12} = \delta(\Delta^{\omega}_{k;12}) = \delta(\omega_k - \omega_1 - \omega_2)$. Equation 27 states that in the long-time limit the only interactions that are effective are due to triads that satisfy the *resonance* conditions imposed by the factors $\delta^{\omega}_{k;12}\delta^{k}_{k;12}$, i.e., satisfying the conditions,

$$\Delta^{k}_{k;12} = k - k_1 - k_2 = 0, \tag{29a}$$

$$\Delta^{\omega}_{k;12} = \omega(k) - \omega(k_1) - \omega(k_2) = 0, \tag{29b}$$

equivalent to the "maximal" effectiveness of nonlinear interaction (see discussion of equations **??**). Whether or not equation 13 has resonant triads depends on the linear properties of the physical system. The resonance conditions play an important role in the stochastic theory [e.g., Zakharov et al., 1992, Nazarenko, 2011, Anenkov and Shrira, 2018].

3.3. **Conservation laws for the kinetic equation.** The dynamical equation 13 has one integral of motion, the Hamiltonian 11. The kinetic equation 27 has its own set of integrals. Let Q be the *physical-space* density of an extensive quantity,

$$Q(t) = \int \mathscr{q}(k,t)dk = \int \rho(k)n(k,t)dk. \tag{30a}$$

where $\rho(k)$ does not depend on time. Examples of such quantities are the energy E with densities $\mathscr{e} = \omega(k)n$, and momentum M with densities $\mathscr{m} = kn$. Using the kinetic equation, the time derivative of Q is

$$\dot{Q} = \pi \int_{-\infty}^{\infty} \rho_k \left(\mathscr{R}_{k;12} - \mathscr{R}_{1;k2} - \mathscr{R}_{2;k1}\right) dk. \tag{30b}$$

The quantity Q is conserved if $\dot{Q} = 0$. In general, the conservation equation 31 may also be recast as a continuity (transport) equation

$$\partial_t \mathscr{q}(k,t) + \partial_k F_{\mathscr{q}}(k,t) = 0. \tag{30c}$$

where $\mathscr{F}_{\mathscr{q}}$ is the spectral flux of $\mathscr{q}$. Comparing equations 31 and 30c, one can write

$$\mathscr{F}_{\mathscr{q}}(k) = -\int_0^k dk\rho_k \left(\mathscr{R}_{k;12} - \mathscr{R}_{1;k2} - \mathscr{R}_{2;k1}\right). \tag{30d}$$

If the density $\mathscr{q}(k,t)$ is stationary then $\partial_t \mathscr{q} = \partial_k F_{\mathscr{q}} = 0$, Q is conserved, and the spectral $\mathscr{q}$-flux is constant.

Such conserved quantities exist. For example, if $|V_{k;12}|^2$ is invariant to permutations of indices, relabeling in equation 30b $1 \leftrightarrow k$ in $\mathscr{R}_{1;k2}$ and $2 \leftrightarrow k$ in $\mathscr{R}_{2;k1}$ transforms it into

$$\dot{Q} = \pi \iiint_{-\infty}^{\infty} (\rho_k - \rho_1 - \rho_2) |V_{k;12}|^2 J_{k;12}\delta^{k}_{k;12}\delta^{\omega}_{k;12} dk dk_{12}. \tag{31}$$

The product $\Delta^{\rho}_{k;12}\delta^{k}_{k;12}\delta^{\omega}_{k;12}$ cancels if $\rho = \omega(k)$ or $\rho = k$. It is obvious that the energy E and momentum M, defined as

$$E = \int_0^{\infty} \mathscr{e}(k,t)dk = \int \omega(k)n(k,t)dk, \tag{32}$$

$$M = \int_0^{\infty} \mathscr{m}(k,t)dk = \int kn(k,t)dk, \tag{33}$$

are conserved by the kinetic equation.

3.4. **Stationary solutions of the kinetic equation.**

3.4.1. *Thermodynamic equilibrium, Rayleigh-Jeans (RJ) spectra:* A family of stationary spectra for equation 27 is immediately found by inspection. If we set

$$n^{\mathrm{RJ}}(k) = \frac{T}{c_\omega \omega + c_k k}, \tag{34}$$

where T, c_ω and c_k are constants, the integrand $J_{k;12}\delta^k_{k;12}\delta^\omega_{k;12} = 0$ for *all triads*. Moreover, this spectrum corresponds to the equipartition of the quantity Q with density $\mathcal{q}(k) = (c_\omega \omega + c_k k)\, n_k$; for example, if $c_k = 0$, this represents the equipartition of energy. This type of stationary state corresponds to a "detailed balance", where nonlinear interaction cancels for each triad, and $\dot{n} = 0$ regardless of the quantity $\mathcal{q}$ carried by the number of particles n. The spectral $\mathcal{q}$-flux cancels, in equation 30c $F_{\mathcal{q}} = 0$.

Thermodynamic equilibrium states cannot be realized at all scales because equipartition means that the *physical-space* density $Q = \int T dk$ is infinite (a phenomenon known as the ultraviolet catastrophe). However, it might occur if the spectral fluxes "stagnate" near a critical wavenumber k_0, preventing fluxes to infinite wavenumbers. The system will tend to "thermalize", i.e., approach a zero-flux, thermodynamic equilibrium state (a discussion of this "bottleneck" scenario is given in Nazarenko, 2011).

3.4.2. *Kolmogorov-Zakharov spectra.* In non-isolated systems that have sources and sinks of Q well separated in the spectral domain, one would expect non-zero fluxes $F_{\mathcal{q}}$ from the sources to sinks, similar to the hydrodynamic energy cascade described by Richardson and Kolmogorov [Richardson, 1922, Kolmogorov, 1941, Frisch, 1995]. Nonzero stationary solutions of the kinetic equations were found by Zakharov and Filonenko [1967a,b] and are known as the Kolmogorov-Zakharov (KZ) spectra.

At stationarity, the integral on the left-hand side of equation 27 cancels. Under quite general conditions (e.g., if only one physical process is involved), the dispersion relation and the interaction coefficients are homogeneous of degree α and β, respectively:

$$\omega(k) \propto k^\alpha; \quad \omega(\lambda k) = \lambda^\alpha \omega(k); \tag{35}$$
$$V(\lambda k_1, \lambda k_2, \lambda k_3) = \lambda^\beta V(k_1, k_2, k_3).$$

Because we are in a unidimensional system, we are not concerned is isotropy. Look for a solution in the power law form

$$n(k) \propto k^\nu. \tag{36}$$

in equation 27, change the integration variables to (Zakharov transformation)

$$k = \kappa; \quad k_1 = \frac{k^2}{\kappa_1} = \frac{\kappa^2}{k_1}; \quad k_2 = \frac{k\kappa_2}{\kappa_1}. \tag{37}$$

transforms the second to

$$\int_{-\infty}^{\infty} |V_{1;k2}|^2 J_{1;k2}\delta^k_{1;k2}\delta^\omega_{1;k2} dk_{12} = \int_{-\infty}^{\infty} \left(\frac{k}{k_1}\right)^{2+2\beta-\alpha+2\nu} |V_{k;12}|^2 J_{k;12}\delta^k_{k;12}\delta^\omega_{k;12} dk_{23}.$$

Applying a similar transformation to the third term and denoting $x = -(2+2\beta-\alpha+2\nu)$, the kinetic equation becomes

$$\frac{dn_k}{dt} = \pi k^{-x} \int_{-\infty}^{\infty} \left(k^x - k_1^x - k_2^x\right) |V_{k;12}|^2 J_{k;12}\delta^k_{k;12}\delta^\omega_{k;12} dk_{12}. \tag{38}$$

The spectrum $n(k)$ is stationary if (compare with equation 31) either 1) $x = \omega, \nu = -1 - \beta$, corresponding to the conservation of linear energy, or if 2) $x = 1, \nu = \frac{\alpha-3}{2} - \beta$, corresponding to conservation of momentum. Neither is an RJ spectra, since $\nu \neq \alpha$ and $\nu \neq 1$, therefore neither cancels the integrand $\mathscr{R}_{k;12}$. Note that because the Zakharov transformation 37 is singular in $k_1 = 0$ (non-identity transformation) and the integrals in the kinetic equation 27 are not bounded, the validity of the KZ stationary solution is not guaranteed unless the convergence of the integrals is verified.

A simple scaling argument may be used to show that the spectral energy flux is constant in the the case $x = \alpha$. The continuity equation 30c for energy (equation 32) in the spectral domain is

$$\partial_t e + \partial_k F_e = 0,$$

where the energy flux is (see equation 30d)

$$F_e(k) = -\pi \int_0^k dk\omega_k \int_0^\infty \left(\mathscr{R}_{k;12} - \mathscr{R}_{1;k2} - \mathscr{R}_{2;k1}\right) dk_{12}.$$

Scaling all wavenumbers by k, i.e., changing variables $(k, k_1, k_2) \to k(\kappa, \kappa_1, \kappa_2)$ brings the spectral energy flux to the scaled form

$$F_e(k) = -\pi k^{2\nu+2+2\beta} \int_0^1 d\kappa\omega_\kappa \iint_0^\infty \left(\mathscr{R}_{\kappa;12} - \mathscr{R}_{1;\kappa2} - \mathscr{R}_{2;\kappa1}\right) dk_{12} \tag{39}$$

Setting the scaling factor to be independent of k means setting $2\nu + 2 + 2\beta = 0$, which obtains $\nu = -1 - \beta$, i.e., the KZ spectral slope obtained above. A similar argument shows that the second KZ spectrum ($\nu = \frac{\alpha-3}{2} - \beta$) corresponds to constant spectral fluxes of momentum.

3.5. **The three-wave equation.** The three wave equation is a universal model [Weiland and Wilhelmsson, 1977, Craick, 1985, Zakharov et al., 1992] deriving from the dynamical equation 13 by restricting the interaction to a single triad of modes $(\kappa_1, \kappa_2, \kappa_3)$ satisfying the selection criterion $\kappa_3 = \kappa_1 + \kappa_2$

$$a_k = A_1\delta_{k;\kappa_1} + A_2\delta_{k;\kappa_2} + A_3\delta_{k;\kappa_3} = A_1\delta_{k;\kappa_1} + A_2\delta_{k;\kappa_2} + A_3\delta_{k;\kappa_1\kappa_2}. \tag{40}$$

Substituting equation 40 into the dynamical equation 40 obtains equation

$$i\dot{A}_1 = \omega_1 A_1 + V_{3;12}A_3A_2^*, \quad i\dot{A}_2 = \omega_2 A_2 + V_{3;12}A_3A_1^*, \quad i\dot{A}_3 = \omega_3 A_3 + V_{3;12}A_1A_2. \tag{41}$$

Substituting $A_j = b_j e^{i\theta_j}$, with $b_j, \theta_j \in \mathbb{R}$ and $b_j > 0$, obtains the amplitude-phase representation

$$\begin{aligned}
\dot{b}_1 &= V_{3;12}b_2b_3 \sin\Delta^\theta_{3;12}, & \dot{\theta}_1 &= -\omega_1 - V_{3;12}\frac{b_2b_3}{b_1}\cos\Delta^\theta_{3;12},\\
\dot{b}_2 &= V_{3;12}b_1b_3 \sin\Delta^\theta_{3;12}, & \dot{\theta}_3 &= -\omega_2 - V_{3;12}\frac{b_1b_3}{b_2}\cos\Delta^\theta_{3;12},\\
\dot{b}_3 &= -V_{3;12}b_1b_2 \sin\Delta^\theta_{3;12}, & \dot{\theta}_3 &= -\omega_3 - V_{3;12}\frac{b_1b_2}{b_3}\cos\Delta^\theta_{3;12}.
\end{aligned} \tag{42}$$

The system 42 may be reduced to 4 equations and has analytical solution given in terms of Jacobi elliptic functions. Combining the last three equations yields a system of four equations

with four unknowns: the amplitudes $b_j, j = 1, 2, 3$, and the phase $\Delta^{\theta}_{3;12}$

$$\begin{aligned}
\overset{\bullet}{b_1} &= 2V_{3;12} b_2 b_3 \sin \Delta^{\theta}_{3;12}, \\
\overset{\bullet}{b_2} &= 2V_{3;12} b_1 b_3 \sin \Delta^{\theta}_{3;12}, \\
\overset{\bullet}{b_3} &= -2V_{3;12} b_1 b_2 \sin \Delta^{\theta}_{3;12}, \\
\left(\Delta^{\theta}_{3;12}\right)^{\bullet} &= -\Delta^{\omega}_{3;12} - 2V_{3;12}\left(\frac{b_1 b_2}{b_3} - \frac{b_2 b_3}{b_1} - \frac{b_1 b_3}{b_2}\right)\cos \Delta^{\theta}_{3;12}
\end{aligned} \tag{43}$$

Following the Gaussian closure procedure, and assuming the triad is resonant (see equations 29) one obtains the kinetic three-wave equations [e.g., Rabinovich and Trubetskov, 1989]

$$\begin{aligned}
\overset{\bullet}{n_1} &= 2\pi \left|V_{3;12}\right|^2 n_1 n_2 n_3 \left(\frac{1}{n_3} - \frac{1}{n_1} - \frac{1}{n_2}\right), \\
\overset{\bullet}{n_2} &= 2\pi \left|V_{3;12}\right|^2 n_1 n_2 n_3 \left(\frac{1}{n_3} - \frac{1}{n_1} - \frac{1}{n_2}\right), \\
\overset{\bullet}{n_3} &= -2\pi \left|V_{3;12}\right|^2 n_1 n_2 n_3 \left(\frac{1}{n_3} - \frac{1}{n_1} - \frac{1}{n_2}\right),
\end{aligned} \tag{44}$$

where the notation is the same as the one used for the full kinetic equation 27. Because the physical system described by equations 44 comprises only one triad, stationarity conditions degenerate to detailed balance. The RJ spectrum 34 is obviously a solution: direct substitution of expression 34 into equations 44 cancels the factor in parentheses. Energy and momentum are conserved regardless of whether n_j are stationary or not, because the triad is resonant (equations 26)

$$\begin{aligned}
\sum_{j=1,2,3} \omega_j n_j &= -2\pi \left|V_{3;12}\right|^2 n_1 n_2 n_3 \left(\frac{1}{n_3} - \frac{1}{n_1} - \frac{1}{n_2}\right)(\omega_3 - \omega_1 - \omega_2) = 0 \\
\sum_{j=1,2,3} k_j n_j &= -2\pi \left|V_{3;12}\right|^2 n_1 n_2 n_3 \left(\frac{1}{n_3} - \frac{1}{n_1} - \frac{1}{n_2}\right)(k_3 - k_1 - k_2) = 0.
\end{aligned}$$

References

B. V. Alexeev. *Generalized Boltzmann Physical Kinetics*. Elsevier, 2004.

S. Y. Anenkov and V. I. Shrira. Spectral evolution of weakly nonlinear random waves: kinetic description versus direct numerical simulations. *Journal of Fluid Mechanics*, 844:766–795, 2018.

M. C. Baldiotti, R. Fresneda, and C. Molina. A Hamiltonian approach to Thermodynamics. *Annals of Physics*, 373:245–256, 2016.

H. B. Callen. *Thermodynamics*. John Wiley & Sons, Inc., 1960.

A.D.D. Craick. *Wave interactions and fluid flows*. Cambridge University Press, 1985.

U. Frisch. *Turbulence, The legacy of A.N Kolmogorov*. Cambridge University Press, 1995.

A.N. Kolmogorov. The local structure of turbulence in incompressible viscous fluid for very large Reynolds numbers. *Proceedings: Mathematical and Physical Sciences: Turbulence and Stochastic Process: Kolmogorov's Ideas 50 Years On (Jul. 8, 1991)*, 434(1890)(30):9–13, 1941.

D. C. Montgomery and D. A. Tidman. *Plasma Kinetic Theory*. McGraw-Hill New York, 1964.

S.V. Nazarenko. *Wave Turbulence*. Springer, 2011.

A.C. Newell. Lectures on wave turbulence and intermittency. In H. Berestycki and Y. Pomeau, editors, *Nonlinear PDE's in Condensed Matter and Reactive Flows, Proceedings of the NATO Advanced Study Institute on PDE's in Models of Superfluidity, Superconductivity and Reactive Flows Carqese, France 21 June-3 July 1999,*, volume 569 of *NATO Science Series Series C: Mathematical and Physical Sciences*. Springer Science+Business Media, B.V., 1999.

A.C. Newell and B. Rumpf. Wave turbulence and intermittency. *Annual Review of Fluid Mechanics*, 43:59–78, 2010.

A.C. Newell, S.V. Nazarenko, and L. Biven. Wave turbulence and intermittency. *Physica D: Nonlinear Phenomena*, 152-153:520–550, 2001. doi: 10.1016/S0167-2789(01)00192-0.

M. P. Peterson. Analogy between thermodynamics and mechanics. *American Journal of Physics*, 47:488, 1979.

M.I. Rabinovich and C.I Trubetskov. *Oscillations and Waves in Linear and Nonlinear Systems*. Kluwer Academic Publishers, 1989.

S. G. Rajeev. A Hamilton–Jacobi formalism for thermodynamics. *Annals of Physics*, 323:2265–2285, 2008.

L.F. Richardson. *Weather Prediction by Numerical Process*. Cambridge Univ. Press, 1922.

V.S. Vladimirov. *Methods in the theory of generalized functions*. Taylor & Francis, 2002.

J. Weiland and H. Wilhelmsson. *Coherent non-linear interaction of waves in plasmas*. Pergamon Press, 1977.

G. B. Whitham. *Linear and nonlinear waves*. John Wiley & Sons, 1974.

J. J. Wright and D. T. J. Liley. Simulation of electrocortical waves. *Biological Cybernetics*, 72(4): 347–356, 1995.

V. E. Zakharov and N. N. Filonenko. Energy spectrum for stochastic oscillations of the surface of a liquid. *Soviet Physics Doklady*, 11:881–884, 1967b.

V.E. Zakharov. Statistical theory of gravity and capillary waves on the surface of a finite-depth fluid. *European Journal of Mechanics, B/Fluids*, 18(3):327–344, 1999.

V.E. Zakharov and N.N. Filonenko. Weak turbulence of capillary waves. *Journal of Applied Mechanics and Technical Physics*, 8:37–42, 1967a.

V.E. Zakharov, V.S. L'vov, and G. Falkcovich. *Kolmogorov spectra of turbulence I*. Springer Series in Nonlinear Dynamics. Springer-Verlag, 1992.
